# BNC1 regulates cell heterogeneity in human pluripotent stem cell-derived epicardium

**DOI:** 10.1242/dev.174441

**Published:** 2019-12-13

**Authors:** Laure Gambardella, Sophie A. McManus, Victoria Moignard, Derya Sebukhan, Agathe Delaune, Simon Andrews, William G. Bernard, Maura A. Morrison, Paul R. Riley, Berthold Göttgens, Nicolas Gambardella Le Novère, Sanjay Sinha

**Affiliations:** 1 Wellcome Trust – Medical Research Council Cambridge Stem Cell Institute, Department of Medicine, University of Cambridge, Cambridge CB2 0AZ, UK; 2 Department of Haematology, Wellcome Trust – Medical Research Council Cambridge Stem Cell Institute, University of Cambridge, Cambridge CB2 0AZ, UK; 3 The Babraham Institute, Cambridge CB22 3AT, UK; 4 Department of Physiology, Anatomy and Genetics, University of Oxford, Oxford OX1 3PT, UK; 5 aSciStance, Cambridge CB10 1PF, UK

**Keywords:** Epicardium, Human pluripotent stem cells, Heart development, Regenerative medicine, BNC1, Single cell

## Abstract

The murine developing epicardium heterogeneously expresses the transcription factors TCF21 and WT1. Here, we show that this cell heterogeneity is conserved in human epicardium, regulated by BNC1 and associated with cell fate and function. Single cell RNA sequencing of epicardium derived from human pluripotent stem cells (hPSC-epi) revealed that distinct epicardial subpopulations are defined by high levels of expression for the transcription factors BNC1 or TCF21. WT1^+^ cells are included in the BNC1^+^ population, which was confirmed in human foetal hearts. THY1 emerged as a membrane marker of the TCF21 population. We show that THY1^+^ cells can differentiate into cardiac fibroblasts (CFs) and smooth muscle cells (SMCs), whereas THY1^−^ cells were predominantly restricted to SMCs. Knocking down BNC1 during the establishment of the epicardial populations resulted in a homogeneous, predominantly TCF21^high^ population. Network inference methods using transcriptomic data from the different cell lineages derived from the hPSC-epi delivered a core transcriptional network organised around WT1, TCF21 and BNC1. This study unveils a list of epicardial regulators and is a step towards engineering subpopulations of epicardial cells with selective biological activities.

## Abstract (precis)

**Highlighted Article:** Single cell analysis of human pluripotent stem cell-derived epicardium revealed that epicardial heterogeneity is regulated by the transcription factor BNC1 and separates the cells into two populations with different properties.

## INTRODUCTION

The epicardium is an epithelium covering the heart, which is essential for normal cardiac development. Epicardial cells originate from the pro-epicardial (PE) organ, an outgrowth of non-cardiac, coelomic and proliferative cells located between the heart and the liver (Smits et al., 2018). During embryonic life, the epicardium provides signals for proliferation, survival and maturation to the cardiomyocytes. In return, the myocardium provides signals inducing proliferation and epithelial-to-mesenchymal transition (EMT) in the epicardium. The mesenchymal cells derived from the epicardium (EPDCs) invade the myocardium and become mainly cardiac fibroblasts (CFs) and coronary smooth muscle cells (cSMCs). Surgical ablation of the PE organ leads to cardiac developmental abnormalities (Gittenberger-De Groot et al., 2000).

In the adult, the epicardium is quiescent. It becomes reactivated after ischemic injury but produces EPDCs that are less efficient at migrating and differentiating than their embryonic counterparts. They produce signals that activate the resident CFs, inducing fibrosis but no myogenesis. Some animals, such as the zebrafish and the neonatal (but not adult) mouse, can fully regenerate the heart (Poss et al., 2002; Chablais et al., 2011; Porrello et al., 2011). The adult epicardium is necessary for this regeneration potential (Schnabel et al., 2011). For example, after injury in zebrafish, PDGF signalling is activated in the newly produced EPDCs allowing them to proliferate and contribute to new coronary vessels (Kim et al., 2010). The regenerating epicardium also secretes the cytokine Cxcl12a, which directs the migration of proliferating cardiomyocytes towards the wound. This migration is altered by blocking Cxcr4b, the receptor for Cxcl12a (Itou et al., 2012). A better knowledge of human epicardium could explain and allow us to circumvent the regenerative limitations of the adult heart (Masters and Riley, 2014; Risebro et al., 2015; Smits et al., 2018).

A number of genes expressed in the epicardium that have key regulatory roles have been described. The most important groups for development and function are signalling molecules and growth factors belonging to the FGF, TGF, PDGF, WNT and other families, cell-to-cell adhesion molecules such as E-cadherin or zonula occludens-1 and transcription factors (Braitsch and Yutzey, 2013). The best characterised markers in the epicardial literature are two transcription factors: Wilms' tumor 1 (WT1) and transcription factor 21 (TCF21). TCF21 is a basic helix-loop-helix transcription factor essential for the determination of the CF lineage (Acharya et al., 2012). WT1 is a zinc-finger transcription factor essential for EMT (Martínez-Estrada et al., 2010; von Gise et al., 2011). Interestingly, they are both already expressed in the PE cells, downregulated in the adult epicardium, and reactivated after myocardial ischemic injury.

Previous studies suggested that the epicardium comprises different cell types, with distinct functions (Simões and Riley, 2018; Smits et al., 2018; Weinberger et al., 2018). However, little is known about the molecular regulation of this cellular heterogeneity, except that WT1 and TCF21 are heterogeneously expressed within the mouse epicardium (Braitsch et al., 2012). Identifying the molecular signatures of the various epicardial cells may unveil their different functions. Because a better understanding of the epicardium could pave the way to treating the adult heart following injury, we and others have recently developed and validated protocols to generate *in vitro* models of human developing epicardium from human pluripotent stem cells (hPSC-epi) (Witty et al., 2014; Iyer et al., 2015; Bao et al., 2017; Guadix et al., 2017; Zhao et al., 2017). We hypothesised that analysing gene expression at the single cell level in our system will provide key insights into the molecular and functional regulation of the different human epicardial cell populations.

## RESULTS

### Molecular cell heterogeneity in hPSC-epi and human foetal epicardial explant culture

**Fig. 1.**
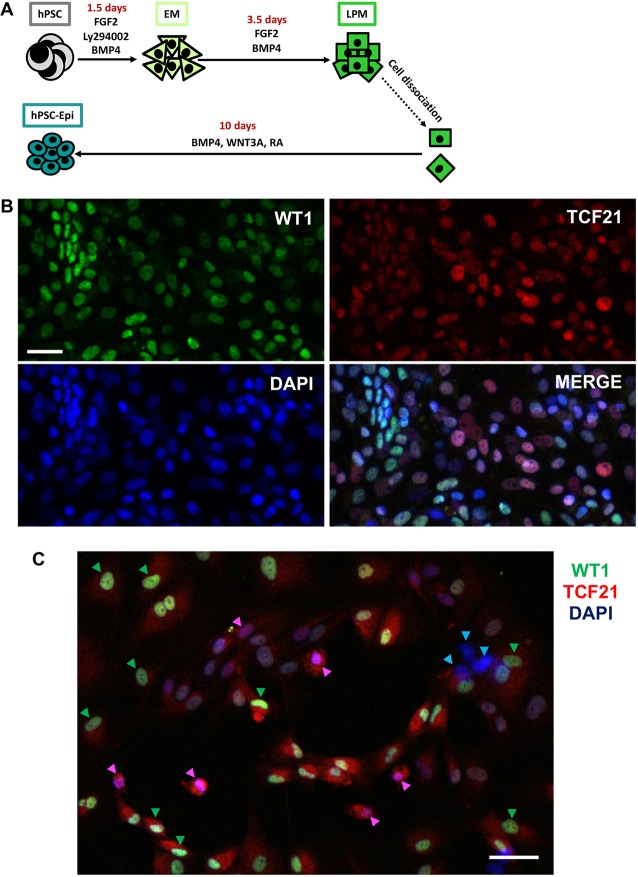
**Heterogeneous expression of TCF21 and WT1 in developing human epicardial cells.** (A) Schematic of the hPSC-epi differentiation protocol. EM, early mesoderm; LPM, lateral plate mesoderm; RA, retinoic acid. (B) Detection of WT1 and TCF21 by immunofluorescence in hPSC-epi. (C) Detection of WT1 and TCF21 by immunofluorescence in epicardial explant cultures from embryonic human heart at 8 weeks. Blue arrowheads point towards double-negative cells, pink and green ones towards TCF21 and WT1 single-positive cells, respectively Scale bars: 20 µm (B); 50 µm (C).

First, we determined the extent of epicardial marker heterogeneity in hPSC-epi cultures. Because both antibodies suitable for the detection of TCF21 and WT1 in human cells were rabbit in origin, we were previously limited to a flow cytometry strategy in which the presence of double-positive cells in the hPSC-epi was indirectly estimated (Iyer et al., 2015). In the present study, we differentiated the hPSC-epi according to the protocol previously published (Fig. 1A). Then, we co-immunostained using an anti-TCF21 antibody plus an Alexa 568-conjugated secondary with sequential application of an anti-WT1 antibody directly conjugated to Alexa 488. This confirmed a clear heterogeneity in the hPSC-epi (Fig. 1B) with single- and double-positive cells. To validate the *in vitro* hPSC-derived model, we generated explant cultures of primary epicardium from 8 week human foetal hearts; co-immunostaining revealed similar heterogeneity in the foetal explants to that observed in the hPSC-derived cells (Fig. 1C). We then sequenced the transcriptome of the hPSC-epi at single cell resolution in order to characterise precisely the molecular heterogeneity of these cells and to determine its physiological regulation and functional relevance.

### scRNA-seq revealed *WT1*, *TCF21* and *BNC1* as indicators of hPSC-epi functional heterogeneity

**Fig. 2.**
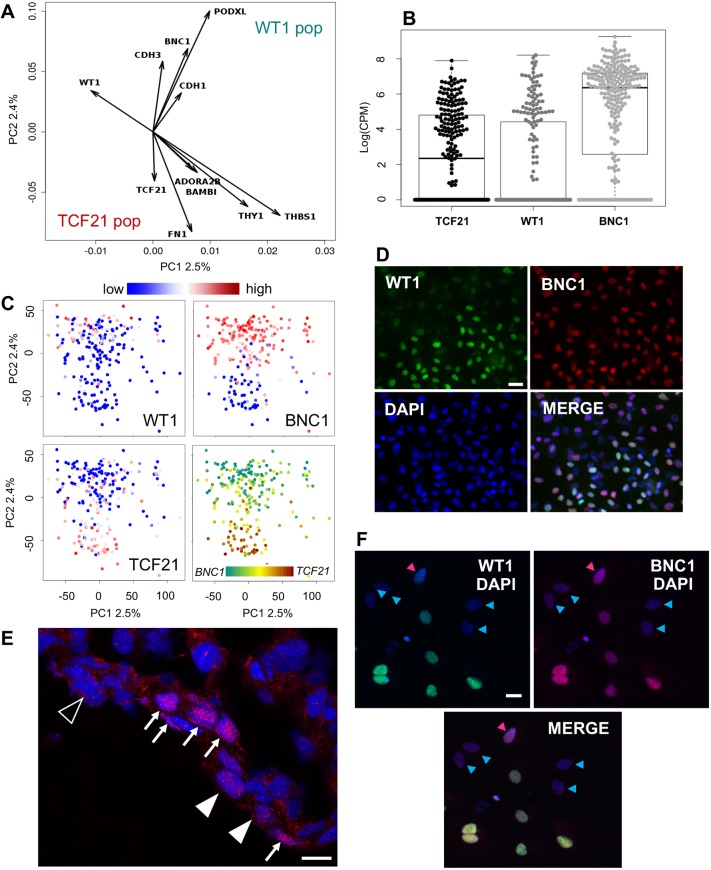
**Characterisation of hPSC-epi heterogeneity by scRNA-seq.** (A) Principal component analysis of the gene expression in hPSC-epi cells, showing some of the main gene influences on PC2. (B) Distribution of expression of TCF21, WT1 and BNC1 in all epicardial cells (232). The numbers of cells for which no expression is detected are 105, 154 and 44, respectively (represented by the thick line at the bottom of the graph). Boxes represent the inter-quartile range (IQR) between quartile 1 (Q1=25%) and quartile 3 (Q3=75%); whiskers represent Q1-1.5×IQR and Q3+1.5×IQR. (C) Principal component analysis of the epicardial cells, coloured by the expression of TCF21, WT1 and BNC1 (see key above), showing the strong alignment with PC2. The lower-right panel presents the overlap of TC21 (in red) and BNC1 (in turquoise) showing that their expression is exclusive. (D) WT1 and BNC1 detected by immunofluorescence in hPSC-epi. (E) BNC1 distribution in human epicardium at 8 weeks pc. Arrows point towards high-expressing cells, filled arrowheads towards low-expressing cells and empty arrowheads to negative cells. (F) WT1 and BNC1 detected by immunofluorescence in epicardial explant cultures from embryonic human heart at 8 weeks pc. The pink arrowheads point toward a single BNC1-positive cell, the blue ones towards double-negative ones. The other cells displayed on the images are double positive. Scale bars: 30 µm (D); 9 µm (E); 20 µm (F).

Using a Smart-Seq2-based protocol previously used to analyse mouse embryonic cells (Scialdone et al., 2016), we obtained high-quality transcriptomes for a total of 232 hPSC-epi single cells. We examined the variation of *TCF21* and *WT1* expression in the population using single cell RNA sequencing (scRNA-seq). As we were using a monolayer of cells obtained from a simple *in vitro* differentiation protocol, we expected subtle levels of heterogeneity in the sequencing data. Indeed, in a principal component analysis (PCA), the first two components only absorbed 2.5% and 2.4% of the variance, respectively. Moreover, the subsequent Eigen values were much smaller, and 195 components were needed to absorb 90% of the variance. The strongest loadings of *TCF21* and *WT1* were on the second component (PC2). Over-representation analyses using the 100 genes with strongest negative and positive PC2 loadings defined two different molecular signatures on the *TCF21* and *WT1* sides. Among the top genes on the *TCF21* side (Fig. 2A), the strongest is coding for fibronectin (FN1), with others coding for thrombospondin (THBS1), THY1, CDH7, BAMBI and adenosine receptor 2B (ADORA2B) (Fig. S1). On the *WT1* side, the strongest is coding for the podocalyxin (PODXL), with others coding for basonuclin (BNC1, second strongest positive loading on PC2), P-cadherin (cadherin 3; CDH3) and E-cadherin (cadherin 1; CDH1).

The distribution of expression for *TCF21*, *WT1* and *BNC1* was bimodal, with a large population of cells showing no expression at all (106, 154 and 45 cells, respectively) (Fig. 2B). It is likely that some of those ‘zeroes’ are dropouts (Andrews and Hemberg, 2019). However, the locations of those cells on the PCA plot suggest that they are not randomly distributed and most of them, at least when it comes to *TCF21* and *BNC1* expression, reflect true subpopulations. Twenty-seven cells (12%) expressed all three markers. Colouring the PCA plot with *TCF21*, *WT1* and *BNC1* expression clearly shows two populations segregated along PC2 (Fig. 2C). With a few exceptions, *WT1*-expressing cells form a subpopulation of *BNC1*-expressing cells. Cells strongly expressing *BNC1* are mostly devoid of *TCF21* expression (see also Fig. S2A). Finally, immunofluorescence detection of BNC1 and WT1 confirmed the correlation between the high level of WT1 and high level of BNC1 and the inclusion of WT1^+^ cells in the BNC1^+^ population in the hPSC-epi (Fig. 2D). Immunostaining of foetal human heart sections at 8 weeks pc demonstrated the expression of BNC1 in the epicardium and confirmed a heterogeneous distribution of the protein (Fig. 2E). Immuno-staining of human embryonic epicardial explants from human hearts at 8 weeks post-conception (pc) confirmed co-location of WT1 and BNC1 (Fig. 2F).

*BNC1* and *TCF21* are not just markers of two subpopulations; they also reflect the state of the entire transcriptome. We computed the Pearson correlation of *BNC1*, *WT1* and *TCF21* expression with all the expressed and variable genes (Fig. S2B). Comparing these correlations, we observe that if the expression of a gene correlates with *TCF21* expression, it does not correlate with *BNC1* expression (Pearson correlation of −0.454). By contrast, if the expression of a gene correlates with that of *WT1*, it also tends to correlate with *BNC1* expression (Pearson correlation of 0.293).

### The hPSC-epi is composed of a BNC1 and a TCF21 population

**Fig. 3.**
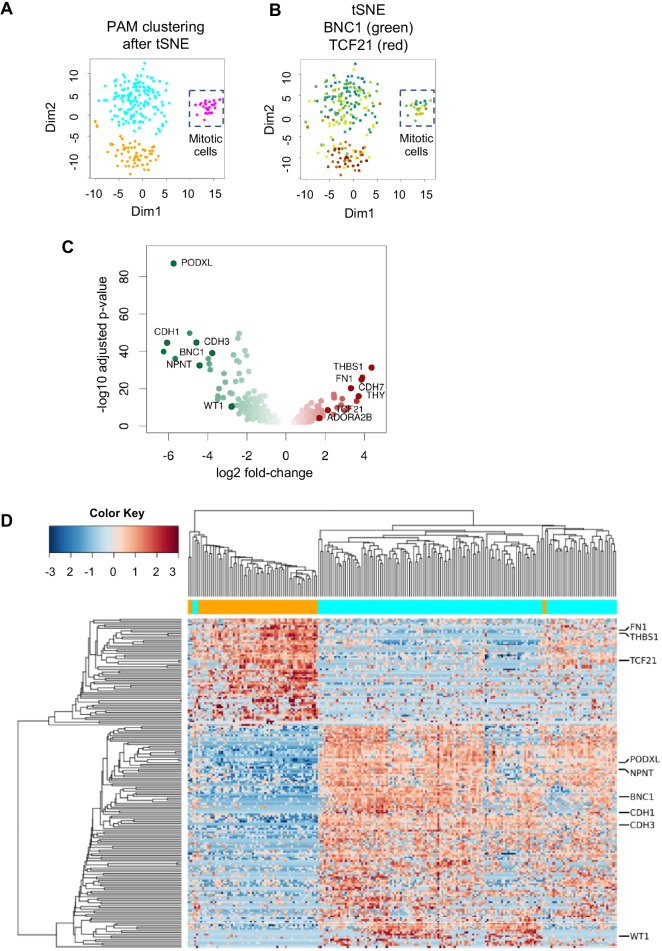
**Transcriptomes of BNC1^high^ and TCF21^high^ subpopulations.** (A) tSNE of all hPSC-epi cells, followed by a clustering using partition around medoids (PAM). (B) Expression of TCF21 (red) and BNC1 (green) showing that the main clusters contain either BNC1 or TCF21 cells whereas the smallest cluster presents a mix of them. (C) Differential expression analysis between the two main clusters showing the amplitude of changes and their significance. Genes of specific interest for epicardium function or this study are highlighted. (D) Heat plot of the most differentially expressed genes between the two main clusters showing that the clustering based on those genes reflects the division based on the whole transcriptome. Columns are cells, rows are genes. The orange and cyan bar specifies which cluster shown in A the cell belongs to.

Cell subpopulations of hPSC-epi cannot be solely based on the expression of *TCF21* and *BNC1* because of the presence of dropouts – genes for which expression is measured as 0 not because there is no mRNA, but because the mRNA was not reverse-transcribed. Instead, we generated cell similarities using t-distributed stochastic neighbour embedding (t-SNE), exploring different parameter values. The lowest Kullback–Leibler divergence (a measure of how well the t-SNE distances represents the actual distances in genome space) corresponded with final distributions in three groups of cells. We attributed cells to each group using a partition around medoid approach, resulting in three clusters of different sizes (146, 62 and 24 cells; Fig. 3A). The largest cluster expressed considerably more *BNC1* than *TCF21*, the intermediate showed the opposite, and the smallest cluster comprised both types of cells (Fig. 3B). We used DESeq2 to compare gene expression between each pair of clusters. Enrichment analysis on the resulting gene sets showed that the small cluster exhibits a clear signal for mitosis (Table S1). When we corrected for a cell-cycle component with the single cell latent variable model (Buettner et al., 2015; Andrews and Hemberg, 2019) before running t-SNE and clustering, the small cluster disappeared, suggesting it was not a true subpopulation. To avoid confusion, these 24 mitotic cells were omitted in further analyses.

### Molecular signature of BNC1^high^ and TCF21^high^ populations

Differential gene expression analysis revealed that 2494 genes were differentially expressed between the largest clusters (Fig. 3A, cyan and orange): 1454 higher in the *BNC1*^high^ cells and 1040 higher in the *TCF21*^high^ ones (Table S2). In addition to an enrichment of 13.6-fold in *BNC1* expression, the *BNC1*^high^ cells exhibited 3.6 times more *WT1* than did *TCF21*^high^ cells (Fig. 3C). Genes encoding podocalyxin, E-cadherin and P-cadherin were also strongly enriched confirming the positive loading of PCA component 2. In addition to 4.4-fold more *TCF21* expression, the *TCF21*^high^ cells showed enrichments for the markers found in the negative loading of PCA component 2. Clustering the cells using 142 strongly expressed (base mean above 100), very significantly (adjusted *P*-value lower than 10^−5^) and strongly enriched (enrichment over 2-fold) genes, reproduced the clustering based on the whole genome (Fig. 3D). This means that the most differentially expressed genes are a good representation of the whole transcriptional landscape, providing confidence that the genes significantly differentially expressed between *TCF21*^high^ and *BNC1*^high^ are valid markers to separate the two populations.

The top transcription factors, plasma membrane proteins and secreted factors upregulated in *BNC1*^high^ and *TCF21*^high^ populations are listed in Table 1. Some of these genes encode for proteins that had already been flagged in the literature as potential regulators of epicardial function in the embryonic or adult diseased heart. The most overexpressed diffusible factor in *BNC1*^high^ cells was nephronectin (*NPNT*). Nephronectin is the functional ligand of integrin alpha-8/beta-1, which is overexpressed in the *TCF21*^high^ population, suggesting cross-talk between the two populations. A web application that enables running PCA, t-SNE and clustering, as well as plotting gene expression, is available at http://www.bioinformatics.babraham.ac.uk/shiny/epicardium/ (Fig. S3).

**Table 1.**
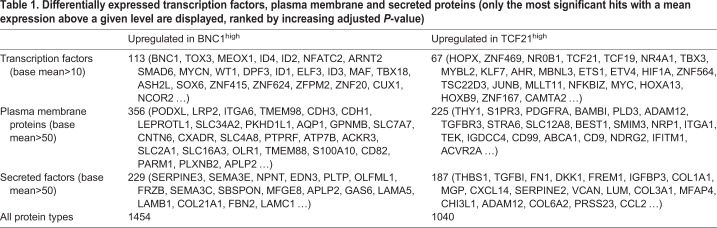
**Differentially expressed transcription factors, plasma membrane and secreted proteins (only the most significant hits with a mean expression above a given level are displayed, ranked by increasing adjusted *P*-value)**

### Gene ontology analysis predicts different functions for *BNC1*^high^ and *TCF21*^high^ populations

**Fig. 4.**
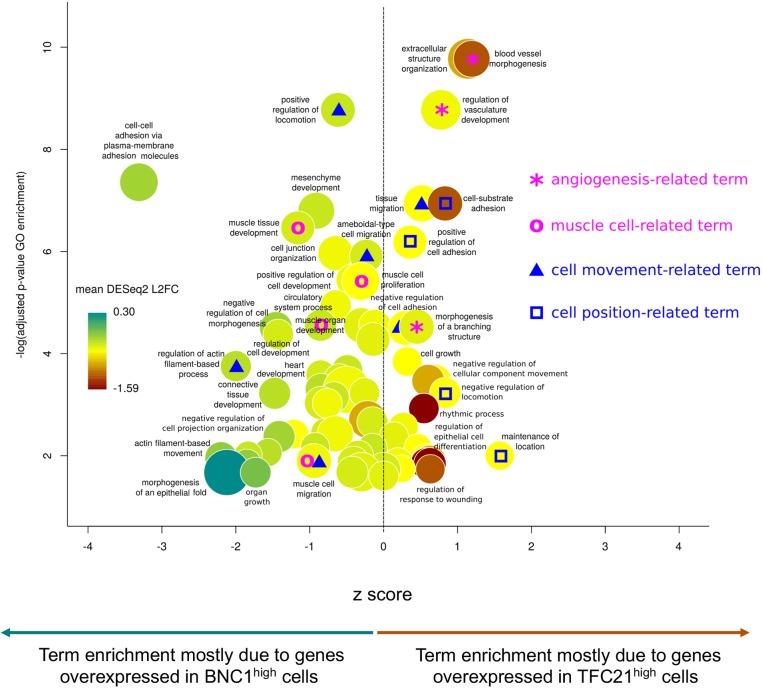
**Predicted tissue and cellular specificities of BNC1^high^ and TCF21^high^ cells.** Results of gene ontology over-representation and gene expression differential analyses. Each bubble represents an over-represented GO term, the disk size being proportional to the enrichment. The *y*-axis presents the significance of the enrichment and the *x*-axis indicates if the term enrichment is mostly due to genes over-expressed in BNC1^high^ cells (negative z-scores) or in TCF21^high^ cells (positive z-scores). Bubble colours show the mean difference of expression, for all the genes annotated by the GO term, between BNC1^high^ cells (turquoise) and TCF21^high^ cells (red).

Gene ontology (GO) over-representation analyses suggested a different phenotypic signature for each population, favouring migration and muscle differentiation for *BNC1*^high^ and adhesion/angiogenesis for *TCF21*^high^. Using the genes differentially expressed between the two populations, we ran GO over-representation analyses using WebGestalt (Table S3). Fig. 4 and Fig. S4 illustrate results of the GO term enrichment, after filtering out the terms related to non-cardiac tissues. Fig. 4 focusses on the terms related to cell and tissue processes whereas Fig. S4 focusses on the terms related to molecular processes. The *BNC1*^high^ population expressed more genes involved in muscle differentiation, migration and cell-cell interaction. In contrast, the *TCF21*^high^ was characterised by adhesion with the term ‘cell-substrate adhesion’ showing high significance and high specificity to this population. Moreover, Fig. 4 and Fig. S4 revealed an angiogenic activity restricted to the *TCF21*^high^ cells. In particular the GO term ‘blood vessel morphogenesis’ showed high significance, the highest z-score, with genes highly specific to *TCF21*^high^ population (Fig. 4). Furthermore, a large number of genes involved in VEGF production were expressed specifically in the *TCF21*^high^ population (Fig. S4).

### THY1 is a membrane marker of the *TCF21*^high^ population enriched in CF potential

**Fig. 5.**
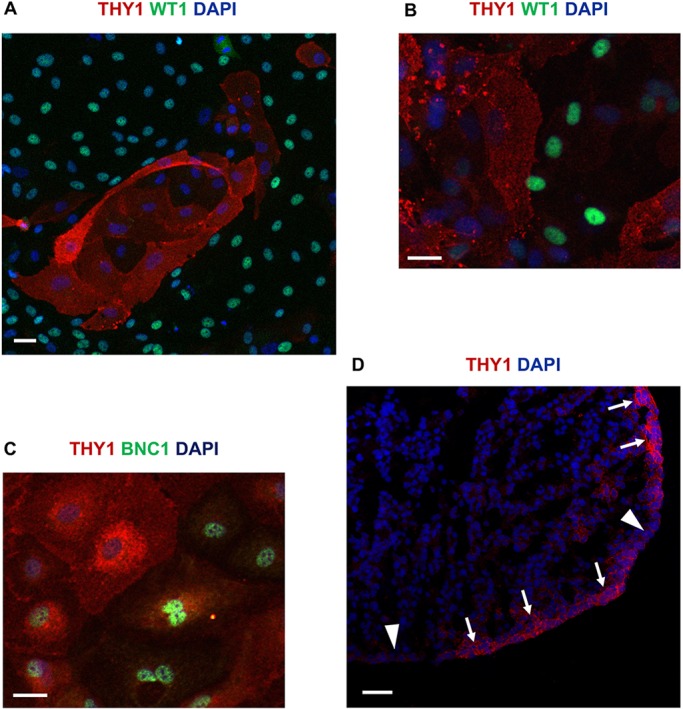
**THY1 expression in epicardium.** (A) Immunofluorescence double labelling for THY1 and WT1 in hPSC-epi. (B,C) Immunofluorescence double labelling in an epicardial explant from an 8 week pc human embryo for THY1 and WT1 (B) or THY1 and BNC1 (C). (D) THY1 immunofluorescence staining on a cryosection of a human heart at 8 weeks pc. The regions of high or low expression of THY1 are indicated by arrows or arrowheads, respectively. Scale bars: 40 μm (A); 20 μm (B,C); 50 μm (D).

**Fig. 6.**
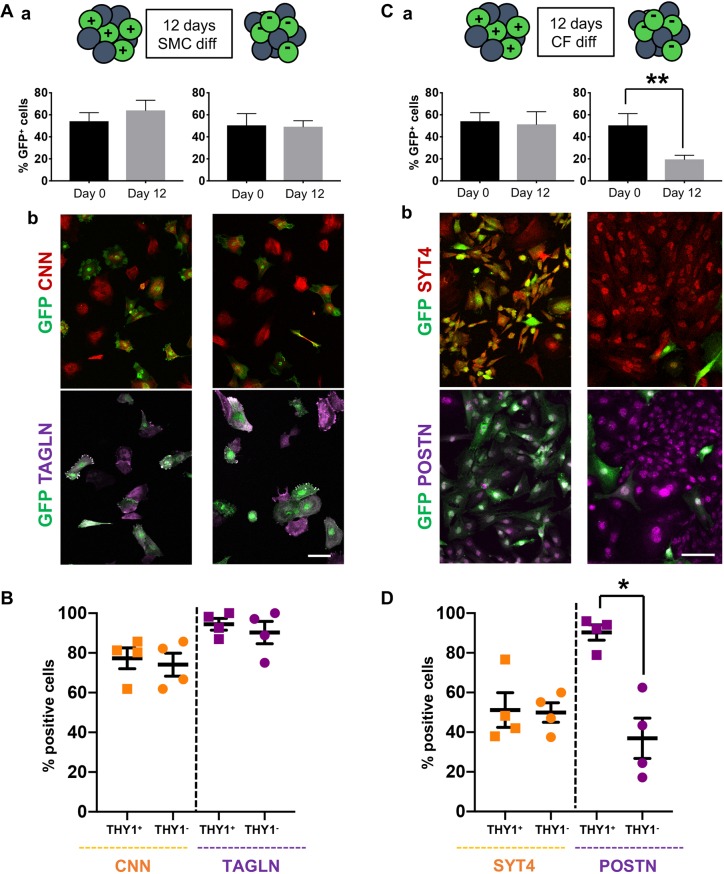
**The THY1^+^ population retains CF potential.** THY1-positive (THY1^+^) and THY1-negative (THY1^−^) cells were magnetically separated from GFP-positive (GFP^+^) hPSC-epi and mixed with regular GFP-negative hPSC-epi in known proportions measured by flow cytometry. (A) After 12 days of differentiation in SMC medium, the cultures were analysed by flow cytometry to establish the percentage of GFP-positive cells still present (Aa, *n*=5) and stained for CNN and TAGLN to confirm the differentiation (Ab). (B) The percentage of CNN^+^ or TAGLN^+^ cells present in the GFP^+^ fraction were quantified in four experiments (an average of 31 and 40 GFP^+^ cells from THY1^+^ and THY1^−^ origin, respectively, were counted in each CNN experiment; an average of 33 and 45 GFP^+^ cells from THY1^+^ and THY1^−^ origin, respectively, were counted in each TAGLN experiment). (C) After 12 days of differentiation in CF medium, the cultures were analysed by flow cytometry to establish the percentage of GFP-positive cells still present (Ca, *n*=5; ratio paired *t*-test performed in Prism 7 from GraphPad) and immunostained for SYT4 and POSTN (Cb). (D) The percentage of SYT4^+^ or POSTN^+^ cells amongst the GFP^+^ population was quantified (*n*=4 and ratio paired *t*-test performed in Prism 7 from GraphPad; an average of 215 and 67 GFP^+^ cells from THY1^+^ and THY1^−^ origin, respectively, were counted in each SYT4 experiment; an average of 2125 and 442 GFP^+^ cells from THY1^+^ and THY1^−^ origin, respectively, were counted in each POSTN experiment). All error bars represent s.e.m. Scale bars: 130 µm (Ab); 80 µm (Cb).

In order to separate the two populations, we searched for specific membrane-associated proteins and cell surface receptors. *THY1* was was expressed at a level 13 times higher in *TCF21*^high^ than in *BNC1*^high^ cells. (Table 1). Immunofluorescence confirmed that the distribution of the protein THY1 was indeed negatively correlated with WT1 in our system (Fig. 5A) and to WT1 and BNC1 in primary human foetal epicardial explants (Fig. 5B,C). As THY1 had not been reported before in the epicardium, we validated its expression on cryosections of human embryonic hearts at 8 weeks pc. Immunofluorescence confirmed a heterogeneous expression of THY1 in the human developing epicardium (Fig. 5D). We used an anti-THY1 antibody to magnetically separate the two epicardial populations from constitutive GFP-expressing (GFP^+^) hPSC-epi and analyse their capacity to respond to differentiation signals. To analyse the developmental potential of each population under normal conditions, we mixed each fraction with non-fractionated GFP-negative (GFP^−^) hPSC-epi cells in equal proportions and recorded the exact percentage of GFP^+^ cells at day (D) 0 by flow cytometry. The two mixes made of [unfractionated GFP^−^ hPSC-epi and GFP^+^ THY1^+^ hPSC-epi] or [GFP^−^ hPSC-epi and GFP^+^ THY1^−^ hPSC-epi] were separately cultured in differentiation medium for SMCs and CFs. The SMC and CF differentiation media, established previously (Iyer et al., 2015), were made of chemically defined medium (CDM) supplemented with TGFβ and PDGF−BB or VEGFB and FGF2, respectively. When subjected to SMC differentiation, the proportion of GFP^+^ cells remained unchanged with both THY1 fractions (Fig. 6Aa, *n*=5). We stained the cells for two well-characterised markers of the SMC lineage, calponin (CNN) and transgelin (TAGLN) (Fig. 6Ab). Amongst the GFP^+^ cells, we quantified, in four experiments, the percentage of cells positive for these SMC markers and found a similar percentage for THY1^+^ and THY1^−^ origin, indicating they all differentiated well into SMCs (Fig. 6B).

In the CF differentiation medium, the proportion of GFP^+^ cells remained unchanged in the culture containing the THY1^+^ population but was reduced by more than half (19.5% versus 50%) in the condition containing the THY1^−^ population (Fig. 6Ca, *n*=5). We concluded that the THY1^−^ hPSC-epi cells did not survive well in response to FGF2 and VEGFB. Reviewing the molecular signature of THY1^+^ and THY1^−^ populations, which coincided with the TCF21^+^ and TCF21^−^ cells, respectively, we noted that NRP1, one of the receptors for VEGFB, is mostly expressed in the THY1^+^ fraction (see Table 1), which could give an advantage to THY1^+^ cells over THY1^−^ in CF conditions.

Furthermore, we immunostained the cultures for synaptotagmin 4 (SYT4) and periostin (POSTN) (Fig. 6Cb). SYT4 has been identified in our *in vitro* differentiation system as upregulated in the hPSC-epi-CF compared with the hPSC-epi or hPSC-epi-SMC (Fig. S5). POSTN is a well-established marker of CF. To assess whether the surviving GFP^+^ hPSC-epi cells coming from the THY1^−^ origin could acquire a CF signature, we quantified, amongst the GFP^+^ cells, the percentage of SYT4- or POSTN-positive cells. There were equivalent numbers of SYT4^+^ regardless of THY1 status (Fig. 6D, *n*=4). However, only 37% of the GFP^+^ cells expressed POSTN with a THY1^−^ origin compared with 90% with a THY1^+^ origin (Fig. 6D, *n*=4). Thus, those Thy1^−^ cells that do survive CF conditions respond poorly to the FGF2+VEGFB stimulus to turn into CF. As only 40% of the THY1^−^ cells survived under CF differentiation conditions and only 37% of the survivors expressed POSTN, in total only 15% of the THY1^−^ isolated cells acquired a CF identity versus 90% for THY1^+^. Given that we used positive selection to isolate the TCF21/THY1^high^ population, it is likely that a number of TCF21/THY1^low^ cells will have been present in the so-called THY1^−^ fraction; we hypothesise that the CF cells generated from the THY1^−^ fraction originated in fact from those TCF21/THY1^low^ cells. Regardless, we can conclude that the THY1^+^ hPSC-epi cells had a higher propensity (at least six times higher with the current data) to become CFs than the THY1^−^ fraction.

### A core epicardial transcriptional network is coordinated by BNC1, TCF21 and WT1

**Fig. 7.**
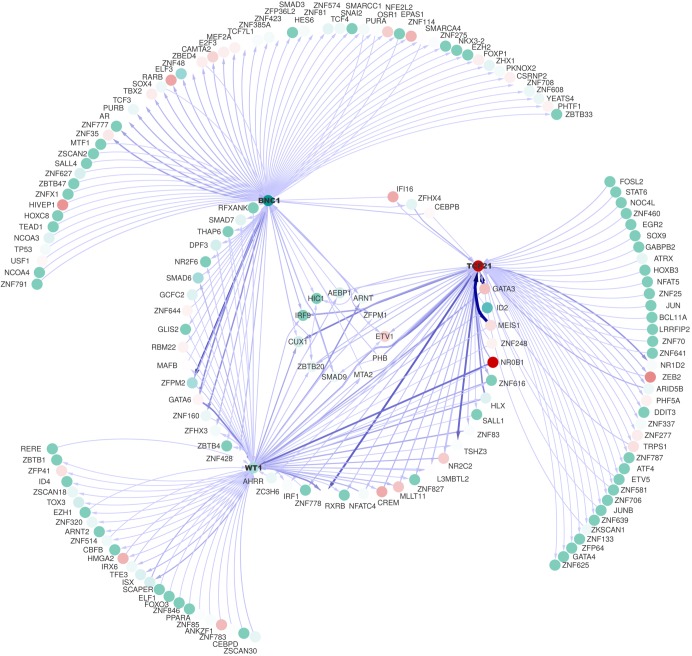
**Core epicardial transcriptional network coordinated by BNC1, TCF21 and WT1.** The network is built using the 100 strongest inferred influences between any of BNC1, TCF21 and WT1 and other transcription factors. The central nodes interact with all three baits, the nodes on the middle circle interact with two of our baits whereas the nodes on the external circle only interact with one bait. Node colours represent the relative expression of the transcription factor in the two populations, turquoise for BNC1^high^ and red for TCF21^high^. The thickness and density of the edges reflect the likelihood of the inferences. Note that because the network is directed, some pairs of nodes are linked by two edges going in opposite directions, although in most cases only one edge passed our threshold.

Network inference methods applied to our system positioned BNC1 as a master regulator, sitting on top of an epicardial regulatory network. To understand better the implications of BNC1, TCF21 and WT1 in the regulation of the epicardial development and function, we inferred a directed transcriptional regulatory network using a combination of methods, context likelihood of relatedness (CLR) and gene network inference with ensemble of trees (GENIE3), as described in the Materials and Methods. The variation in the system was generated by using the bulk sequencing transcriptomic data from different stages of cell development including SMC differentiated from hPSC-derived lateral plate mesoderm (pre-epicardial stage in our system), hPSC-epi, hPSC-epi-CF and hPSC-epi-SMC. We retained the top 100 predicted functional interactions between any transcription factor and each of BNC1, TCF21 and WT1. BNC1 and TCF21 shared three interactors, BNC1 and WT1 shared 17 interactors, and WT1 and TCF21 shared 21 interactors. Eleven transcription factors are interacting with the three baits. The top 100 influences involving *TCF21* showed a balanced picture with 48 influences originating from *TCF21*, and 52 targeting *TCF21*, many influences being bidirectional. The image was similar for WT1. By contrast, 68% of influences involving *BNC1* originated from this gene (Fig. 7, Table 2, Table S4), the imbalance being even more striking in the 50 strongest interactions, where only nine influences targeted *BNC1*. These findings suggest that BNC1 may be a master regulator of epicardial function.

**Table 2.**
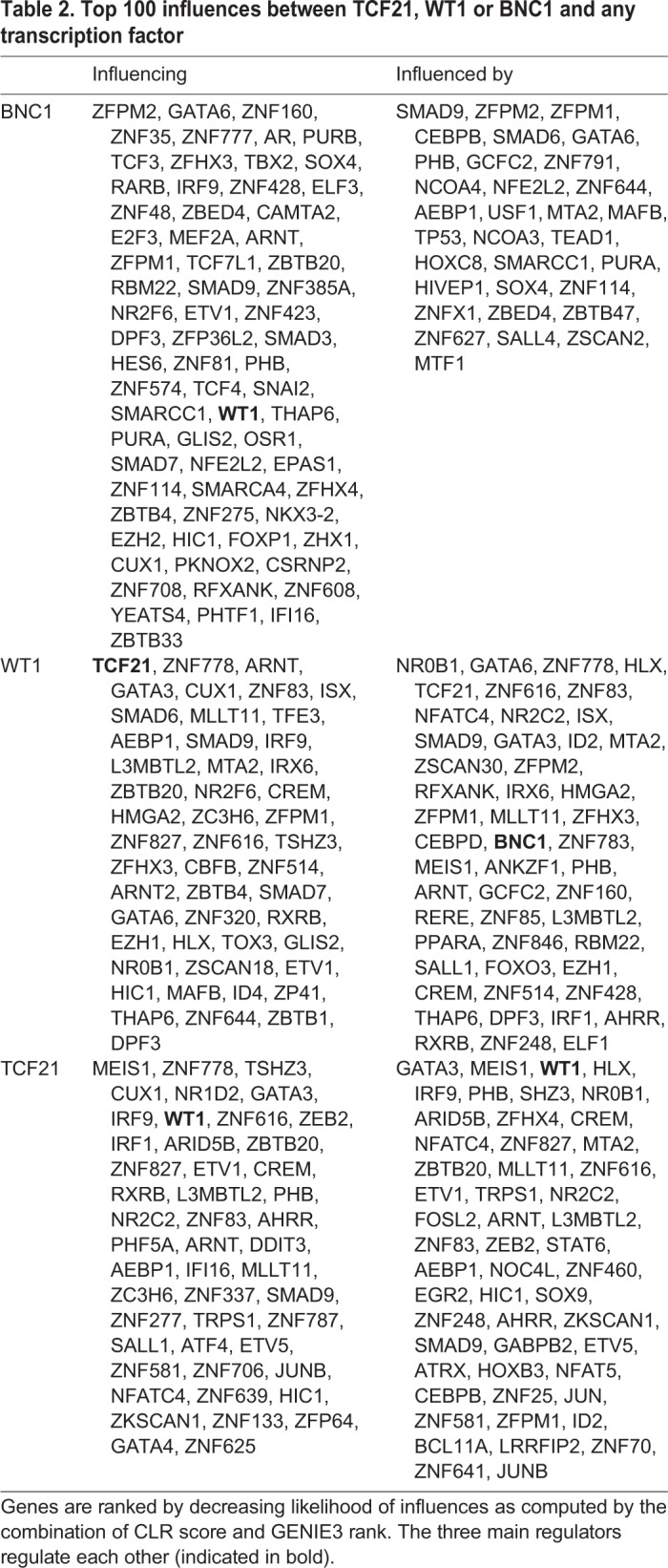
**Top 100 influences between TCF21, WT1 or BNC1 and any transcription factor**

### BNC1 is necessary for epicardial heterogeneity

**Fig. 8.**
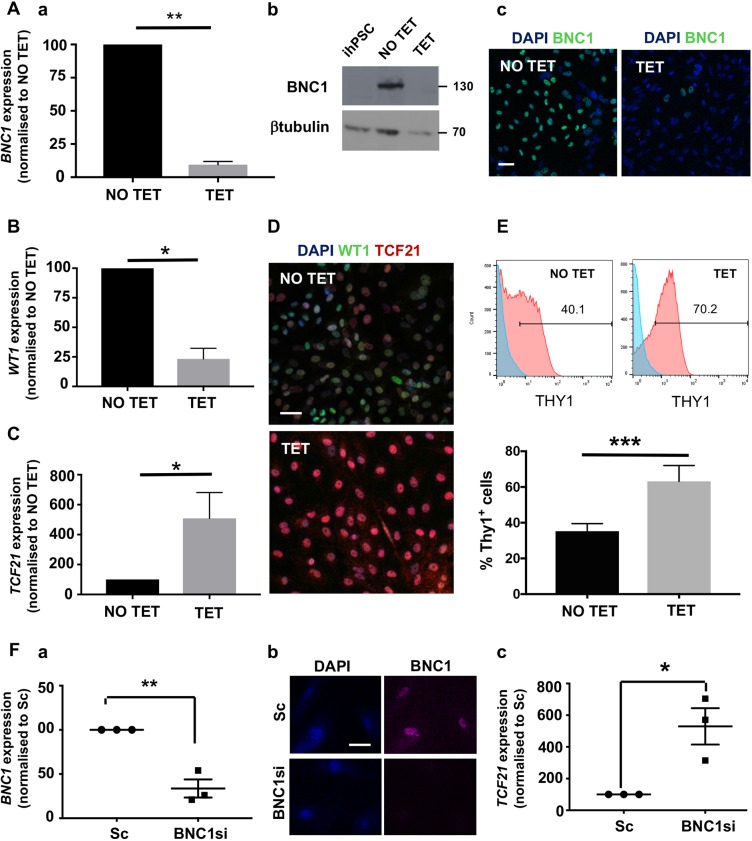
**BNC1 function in developing epicardial cells.** (A) hPSC-epi developed from TET-inducible knockdown hPSC showed more than 90% reduction in *BNC1* RNA under the TET condition (Aa) and 98% reduction at the protein level by western blot (Ab) as also visualised by immunofluorescence (Ac) (*n*=5). (B,C) These cells showed more than 75% reduction of *WT1* RNA (B) and a 5-fold increase in *TCF21* RNA (C). (D,E) When BNC1 is silenced during its development, the hPSC-epi is enriched in the TCF21^high^ population as revealed by double immunofluorescence WT1/TCF21 (D) and THY1 flow cytometry analysis [E; histograms of a representative experiment (top) and recapitulative graph (bottom) of *n*=5; brackets indicate the percentage of positive cells]. (F) BNC1 silencing can be achieved in human foetal primary epicardium using siRNA as shown by RT-PCR (Fa) and immunofluorescence (Fb). The knockdown of BNC1 in human foetal primary epicardium leads to a greater than 5-fold increase in *TCF21* RNA (Fc) (*n*=3). The RT-PCR data shown in Aa, B, C, Fa and Fc were obtained by the quantitative relative standard curve protocol as described in Materials and Methods. RNA measurements were normalised to housekeeper genes porphobilinogen deaminase (*PBGD*) or *GAPDH*. Statistics were performed with Prism 7 from GraphPad with a ratio paired *t*-test. Error bars represent s.e.m. *P<0.05, **P<0.01, ***P<0.001. Scale bars: 40 μm (Ac,D); 20 μm (Fb).

To investigate the function of BNC1 in hPSC-epi, we generated hPSCs that were genetically modified with tetracycline (TET)-inducible shRNA for *BNC1* knockdown. The cells were treated with TET from the last day of the lateral plate mesoderm stage and during the entire differentiation to hPSC-epi. qPCR, western blotting and immunofluorescence showed robust *BNC1* silencing under TET treatment (more than 90% by RT-PCR) (Fig. 8A).

We measured the expression of *WT1* and *TCF21*. *WT1* was downregulated 4-fold (Fig. 8B) whereas *TCF21* was upregulated 6-fold (Fig. 8C) when hPSC-epi was differentiated under a low level of BNC1. Double immunostaining against TCF21 and WT1 confirmed that the low-BNC1 hPSC-epi contained a majority of TCF21^high^ cells (Fig. 8D). As expected, there was also a significant increase in THY1^+^ cell proportions (from 35% to 63%) (Fig. 8E).

We also tested the effect of *BNC1* knockdown in human foetal samples by transfecting primary epicardial cultures derived from foetuses over 10 weeks with small interfering (si) RNA. In line with the results obtained in the hPSC-epi, the silencing of *BNC1* induced a significant (*P*=0.02) 5-fold increase in *TCF21* expression (Fig. 8F).

In conclusion, in the absence of BNC1, the hPSC-epi behaves as a TCF21^high^ population. Therefore, by suppressing the expression of a single transcription factor, we have modified the cell heterogeneity of the hPSC-epi. Thus, we are able to generate a pure TCF21^high^ epicardial population, without requiring sorting methods, as an important step to generating fine-tuned subpopulations of epicardial cells with more specific biological activities.

## DISCUSSION

We have defined the extent of molecular and functional heterogeneity in developing human epicardium by single cell analysis of an hPSC-epi model and validated our findings in human foetal epicardium. We first showed that high levels of expression of TCF21 and WT1 were mutually exclusive. We then identified the transcription factor BNC1 and the membrane protein THY1 as markers of the TCF21^low^ and TCF21^high^ populations, respectively. We discovered that only the TCF21^high^ population can generate CFs. Using a BNC1 knockdown cell model, we demonstrated that BNC1 is essential for the presence of the TCF21^low^ population and required for cellular heterogeneity. Transcriptomics-based network inference predicted the 100 transcription factors most likely to interact with WT1, TCF21 and BNC1, providing several other candidates for the regulation of epicardial function.

BNC1 is a zinc-finger protein and consequently a putative transcription factor but its target genes are still unknown. It is mostly expressed in the basal layers of epidermis, hair follicles and corneal epithelium where it is important for the proliferation of keratinocytes (Zhang and Tseng, 2007). It is abundant in the ovary and testis where it is involved in regulating the proliferation and survival of germ cells (Zhang et al., 2012). It was found expressed in the adult epicardium of the mouse and is downregulated after myocardium injury (Bochmann et al., 2010). We identified for the first time its expression in epicardium during human heart development. Our data suggest that it is an upstream regulator of a transcriptional hierarchy regulating cell identity in the developing epicardium.

Previous studies on epicardium have suggested cellular heterogeneity based on its multifunctionality (reviewed by Simões and Riley, 2018). Yutzey and colleagues demonstrated WT1 and TCF21 heterogeneity of expression by immunocytochemistry on mouse and chick developing heart sections (Braitsch et al., 2012). The Tallquist group have described functional subsets of epicardium based on PDGFRα or PDGFRβ expression, which respectively give rise to CFs or coronary SMCs (Smith et al., 2011). However, the extent of heterogeneity at the single cell level and the molecular regulation of this heterogeneity remain unclear.

Several recent studies have investigated cellular heterogeneity within the heart by scRNA-seq. However, none of these studies was performed with human samples but with mouse and as the entire heart was analysed, the epicardial fraction was usually too small to be adequately studied (DeLaughter et al., 2016; Skelly et al., 2018; Cui et al., 2019). Only two studies, so far, focussed primarily on the epicardium but both were carried out in zebrafish (Cao et al., 2016; Weinberger et al., 2018) with Cao et al. focussing only on TCF21^+^ cells. However, our TCF21^high^ and BNC1^high^ subpopulations seem to be present in Weinberger's data, with our BNC1^high^ cells possibly corresponding to their SEMA3E/PODXL population.

The hPSC-epi system presents the key advantages of *in vitro* systems, particularly advantageous to the study of human biology, such as accessibility for perturbation and analysis, unlimited number of cells, traceability and quality control. However, it is essential to validate the hPSC-epi cells in terms of ability to model events *in vivo*. Comparison between BNC1^high^ and TCF21^high^ in differentiation media showed that only TCF21^high^ cells were able to survive in CF medium whereas both could in SMC conditions. These data are consistent with studies in the mouse by the Tallquist group, which identified TCF21 expression as necessary for differentiation into CFs (Acharya et al., 2012). Our *in vitro* system was further validated by their previously mentioned findings, in which PDGFRα was necessary for CF (Smith et al., 2011) and PDGFRβ for SMC (Mellgren et al., 2008) development. Similarly, in the hPSC-epi, only the TCF21^high^ cells expressed PDGFRA whereas PDGFRB expression was detected in both populations.

Additionally, we have used the system to make predictions about the molecular regulation of epicardial functions, which require further validation. We predicted BNC1 to be a potential master regulator of cell heterogeneity, influencing TCF21 expression and the percentage of cells in the TCF21^high^/THY1^+^ population. We first validated this discovery in primary foetal epicardial cells by downregulating BNC1 with siRNA and measuring the effect on TCF21 expression and the percentage of THY1^+^ cells. Analogous studies *in vivo* using BNC1 gene knockout models in the mouse would further validate the *in vitro* findings presented here.

A powerful application of our hPSC-epi subpopulations may be cell therapy for cardiac repair. Winter and colleagues showed in 2007 that injecting human adult EPDCs generated from an atrial biopsy, into mouse infarcted myocardium, improved heart recovery (Winter et al., 2007). We recently showed that hPSC-epi cells increased cardiac graft size, maturity, vascularisation and cardiac function when added to hPSC-derived cardiomyocytes as combination therapy (Bargehr et al., 2019). Primary human EPDCs from foetal and adult origin display different activation states, which may explain a more rapid foetal EPDC response to environmental cues (Moerkamp et al., 2016). Unlike adult cells, foetal cells progressed through EMT faster, even in the absence of TGFβ signalling, and foetal epithelial EPDCs already had a mesenchymal signature, predisposing them to undergo EMT. Our hPSC-epi also possess a mesenchymal signature (TCF21, THY1, FN1, VIM) and are predictably closer to foetal than adult epicardium (Patterson et al., 2012; DeLaughter et al., 2016; Moerkamp et al., 2016), which may explain why they outperformed adult primary mesenchymal stem cells in engineered heart tissues (Bargehr et al., 2019).

Although cell therapy using hPSC-epi may be beneficial post-myocardial infarction, there is the risk of generating excessive CFs, with subsequent fibrosis leading to ventricular diastolic dysfunction. Consequently, it may be advantageous to separate the cells producing CFs from those producing SMCs. Our work opens the door to this approach by separating the TCF21^high^ from the BNC1^high^ populations. Because THY1 was identified as a membrane marker of the TCF21^high^ cells, we sorted them magnetically using an anti-THY1 antibody. HPSC-epi also generated a restricted TCF21^high^ population following BNC1 knockdown. However, TCF21^high^-derived CFs may have fibrotic potential as well as the possibility of producing deleterious factors such as DKK1 or fibronectin (Wo et al., 2016; Valiente-Alandi et al., 2018). Consequently, the BNC1^high^ population may be more attractive for cell therapy as these cells do not produce CFs, but do express promising factors previously noted for their beneficial properties on cardiomyocytes such as nephronectin (Patra et al., 2012) and milk fat globule-EGF factor 8 (Deng et al., 2017). It will be interesting in future studies to knock down TCF21 to see if we promote the SMC pathway, avoiding a CF fate and potentially deleterious secreted factors. However, the situation with hPSC-epi subpopulations may not be that simple. For example, fibronectin has been reported to have positive or negative effects depending on the context (Wang et al., 2013). Moreover, loss of TCF21 leads to abnormal EPDCs (Braitsch et al., 2012). Furthermore, our bioinformatics analysis suggested that the angiogenic potential of the epicardium resides in the TCF21^high^ population. So instead of using a pure BNC1^high^ hPSC-epi population for cell therapy following myocardial infarction, it may be preferable to engineer a BNC1^high^-enriched population but with attenuated rather than absent CF potential.

Our work has shown that using the hPSC-epi in association with next-generation single cell sequencing methods is a powerful platform to investigate human epicardial heterogeneity and function. To facilitate further analyses, we have developed a publicly available web application, which can display the expression of any gene found expressed in the hPSC-epi overlaid onto a colour-coded tSNE or PCA plot. This tool enables us to determine whether a gene is expressed in the hPSC-epi and whether it is correlated with the expression of other key epicardial genes and will be of significant value to the field.

The next steps will include refining the biological properties of the hPSC-epi subpopulations and extending the investigations of the different pathways identified in this study into *in vivo* systems. The potential of each subpopulation for cardiac repair should also be investigated in animal models of myocardial infarction.

## MATERIALS AND METHODS

### Tissue culture

#### hPSC-derived cells

hPSCs (H9 line, Wicell) were maintained as previously described (Iyer et al., 2015) and tested every 2 months for *Mycoplasma* contamination. hPSC differentiation was performed in CDM-PVA [Iscove's modified Dulbecco's medium (Gibco) plus Ham's F12 NUT-MIX (Gibco) medium in a 1:1 ratio, supplemented with Glutamax-I, chemically defined lipid concentrate (Life Technologies), transferrin (15 μg/ml, Roche Diagnostics), insulin (7 μg/ml, Roche Diagnostics), monothioglycerol (450 μM, Sigma-Aldrich) and polyvinyl alcohol (PVA, 1 mg/ml, Sigma-Aldrich)] on gelatin-coated plates. The cells were first differentiated into early mesoderm with FGF2 (20 ng/ml), LY294002 (10 μM, Sigma-Aldrich) and BMP4 (10 ng/ml, R&D Systems) for 36 h. Then, they were treated with FGF2 (20 ng/ml) and BMP4 (50 ng/ml) for 3.5 days to generate lateral plate mesoderm. The differentiation of lateral plate mesoderm into epicardium (hPSC-epi) was induced by exposure to Wnt3A (25 ng/ml, R&D Systems), BMP4 (50 ng/ml) and retinoic acid (4 μM, Sigma-Aldrich) for 8 to 10 days after dissociation and re-plating of the lateral plate mesoderm cells at a density of 24,000 cells per cm^2^.

Magnetic separation of the THY1^+^ and THY1^−^ hPSC-epi cells was performed using mouse anti-THY1 antibody clone 5E10 (14-0909-82, Thermo Fisher Scientific; 1/100), biotinylated horse anti-mouse IgG antibody (BA-2000, Vector Laboratories; 1/500) and MACS Streptavidin MicroBeads from Miltenyi Biotec following their instructions.

hPSC-epi-SMC and hPSC-epi-CF were derived from hPSC-epi following Iyer et al. (2015). Briefly, after splitting, the hPSC-epi cells were cultured for 12 days in CDM-PVA supplemented with PDGF-BB (10 ng/ml, Peprotech) and TGFβ1 (2 ng/ml, Peprotech) to obtain hPSC-epi-SMC and with VEGFB (50 ng/ml, Peprotech) and FGF2 (50 ng/ml) to obtain hPSC-epi-CF.

#### Primary human cultures

Human embryonic and foetal tissues were obtained following therapeutic pregnancy interruptions performed at Cambridge University Hospitals NHS Foundation Trust with ethical approval (East of England Research Ethics Committee) and informed consent in all instances.

For embryonic epicardial explants, 8-week pc embryonic hearts were harvested and set up under coverslip on gelatin-coated plates (0.1% gelatin for 20 min at room temperature (RT), followed by advanced DMEM F12+10% foetal bovine serum for storage at 37°C) and primary epicardium medium [1:1 mixture of Dulbecco's modified Eagle's medium (DMEM, Sigma-Aldrich) and Medium 199 (M199, Sigma-Aldrich) containing 100 U/ml penicillin, 100 μg/ml streptomycin, 10% heat-inactivated foetal bovine serum (Sigma-Aldrich)]. After a few days, when epicardial cells had started to explant, SB-435142 (Sigma-Aldrich), 10 µM final concentration, was added to the medium.

For primary foetal epicardial cultures, the heart was removed from foetuses over 10 weeks pc. Several patches of the epicardial layer were peeled off with fine dissecting tweezers and set up to grow in a gelatin-coated 12-well tissue culture plate in primary epicardial medium. After 5 days in a humidified incubator at 37°C and 5% CO2, the growing cells were dissociated with TrypLE Express Enzyme (Life Technologies) and re-plated in primary epicardial medium supplemented with SB-435142 10 μM final concentration. Cells were maintained in the same conditions and passaged 1:2 when confluent.

For primary human foetal fibroblasts, the foetal hearts were harvested at 8-9 weeks pc, cut into small pieces and digested with collagenase (collagenase IV, Life Technologies, 17104019) at 0.25% in Dulbecco's PBS (DPBS; Thermo Fisher Scientific), for 30 min at 37°C with occasional re-suspension. Digested tissues were pushed through a 40 µm cell strainer, washed twice in DPBS and then incubated a further 10 min at 37°C in TrypLE Express Enzyme to obtain a cell suspension. Cells were seeded at 1.2×10^7^ cells per 75 cm^2^ on gelatin-coated plates in DMEM (Sigma-Aldrich) supplemented with 10% foetal calf serum (Sigma-Aldrich)+1 ng/ml FGF2 for 20 min at 37°C. At that stage, only fibroblasts had time to adhere. The medium was refreshed in order to remove all the other cell types.

### RNA sequencing

#### Single cell sequencing

Single cells were sorted by flow cytometry into individual wells of a 96-well plate containing lysis buffer [0.2% (v/v) Triton X-100 and 2 U/μl SUPERaseIn RNase Inhibitor (Invitrogen)] and stored at −80°C. Single cell libraries for RNA sequencing were prepared using the Smart-seq2 protocol (Picelli et al., 2014), whereby 21 cycles were used for the cDNA library pre-amplification. The Illumina Nextera XT DNA sample preparation kit and Index Kit (Illumina) were used for cDNA tagmentation and indexing. Library size and quality were checked using an Agilent High-Sensitivity DNA chip with Agilent Bioanalyser (Agilent Technologies). The pooled libraries of 96 cells were sequenced at the Babraham Institute sequencing facility on an Illumina HiSeq2500 at 100 bp per read. We used one lane per plate, resulting in 250,000 to 5,800,000 reads per sample. The quality of the raw data were assessed using FastQC (https://www.bioinformatics.babraham.ac.uk/projects/fastqc/) for common issues including low quality of base calling, presence of adaptors among the sequenced reads or any other over-represented sequences, and abnormal per base nucleotide percentage. FASTQ files were mapped to the *Homo sapiens* genome GRCh38 using HISAT2 (Kim et al., 2015). We removed the 22 samples (over 384) for which either most of the reads (above 97%) were mapped to the ERCC spike-in, probably representing empty wells, without cells, or for which less than 80% of reads were in genes, or for which less than 2% genes were detected. This represented 2-13 samples per 96-well plate. Of the remaining 362 cells, 130 were from the lateral plate mesoderm stage (hPSC-LM) and the 232 others from hPSC-epi. The data have been deposited in NCBI's Gene Expression Omnibus (Edgar et al., 2002) and are accessible through GEO Series accession number GSE122827.

Preliminary analysis using PCA showed that a few cells were isolated, far from most of their grouped siblings. Those cells had fewer reads than others and a low gene count. We therefore removed 36 cells with fewer than 500,000 reads, and expressing fewer than 7000 genes. The expression of genes was quantified using SeqMonk's RNA-Seq pipeline (https://www.bioinformatics.babraham.ac.uk/projects/seqmonk/). Raw read counts aligned with all exons were summed for each gene.

#### Bulk sequencing

Total RNA was extracted from cultures using the RNeasy mini from Qiagen. DNA contamination was removed from the samples using the DNA-free DNA Removal Kit from Ambion (Thermo Fisher Scientific). cDNA synthesis and Illumina libraries were performed using SMARTer Stranded Total RNA-Seq Kit v2 - Pico Input Mammalian kit from Takara Bio. Unless otherwise stated, the data from bulk cultures were produced as for the single cell libraries (see above). We sequenced the 21 libraries on two lanes. The two BAM files of each samples were then merged, resulting in 10 million to 25 million reads per sample. The data have been deposited in NCBI's Gene Expression Omnibus and are accessible through GEO Series accession number GSE122714.

The expression of genes was quantified with SeqMonk's RNA-Seq pipeline using a further DNA contamination correction because a sample exhibited homogeneous read coverage in introns and intergenic regions.

#### Exploration of transcriptomes

For all data analysis except differential expression, the counts were corrected for library size (counts per million reads). Genes displaying fewer than one read per million in all samples were discarded, as were genes for which expression varied by less than 2-fold across all samples. Counts were then normalised using the *rlog* function of the *DESeq2* R package (Love et al., 2014). hPCS-epi came from two different experiments, and we found a clear batch effect, whereas there was no difference between the cells coming from two different plates sequenced on different lanes. We corrected for the culture batch effect with the *combat* function or the *sva* R package.

PCA was performed using the *prcomp* function of the *stats* R package. t-SNE was carried out using the *rtsne* R package (van der Maaten, 2014). Parameters were explored systematically, and better results were obtained with a perplexity of 30, and a maximum of 2000 iterations. Varying the acceleration parameter θ between 0 and 0.5 did not change the results significantly. Clusters were defined by the partitioning around medoids algorithm using the *pam* function of the *cluster* R package.

Differential expression analyses were performed with the *DESeq2* package (Love et al., 2014). Significance was set at a *P*-value adjusted for multiple testing of 0.01. Over-representation analyses were performed using WebGestalt (Wang et al., 2017) and the non-redundant version of Gene Ontology Biological Process branch (Ashburner et al., 2000). The background for DESeq2-related enrichment was the list of all genes expressed in at least one cell. We retained all the terms with a *P*-value adjusted for multiple testing (FDR) under 0.05. The z-score for each GO term was computed as the difference between the number of genes annotated with this term upregulated in TCF21^high^ and those upregulated in in BNC1^high^ divided by the square root of their sum.

#### Network inference

Following the conclusions of the DREAM5 challenge's analysis (Marbach et al., 2012), we used a combination of methods based on different algorithms to infer regulatory networks. We combined the results of CLR (Faith et al., 2007), a mutual-information-based approach providing undirected edges, and GENIE3 (Faith et al., 2007; Huynh-Thu et al., 2010), a tree-based regression approach providing directed edges. Both methods were the best performers in their category at DREAM5. We applied the two methods to transcription factor gene expression coming from the bulk sequencing samples, using filtered CPM as described above. As CLR only provides undirected edges whereas GENIE3 provides directed ones, the results of CLR were all mirrored with an identical score on edges in both directions. Only edges with a positive score in the GENIE3 analysis were used. The intersect between edges present in CLR and GENIE3 results were then ranked according to the product of both algorithms' scores. This only retains edges that have either an extremely high score with one method, or consistent scores with both methods. Subnetworks were extracted using gene lists as seeds, retaining only the first neighbours above a threshold. Visualisation and analysis of the resulting networks was performed using Cytoscape (Shannon et al., 2003).

### Inducible knockdown (psOPTIkd)

#### Design and annealing of shRNA oligonucleotides

Oligonucleotides were designed by using the TRC sequence from Sigma-Aldrich and are shown in Table S5. Hairpin A was selected as a validating hairpin as it was demonstrated to work previously in downregulating *B2M* expression. The oligonucleotides were annealed according to the protocol supplied by Bertero et al. (2016, 2018) and then ligated into the cut psOPTIkd vector using T4 ligase for 2 h at RT. The ligation mix was transformed into alpha select competent cells (BioLine) according to the manufacturers' directions. The transformations were plated onto LB agar plates containing ampicillin before colony PCR screening of transformants.

#### Colony PCR of transformants

Transformants grown on LB agar plates were picked in the morning after plating for colony PCR. AAVsingiKD forward (CGAACGCTGACGTCATCAACC) and reverse (GGGCTATGAACTAATGACCCCG) primers were used; thermocycling conditions were as follows: 95°C for 5 min, then 35 cycles of 95°C for 30 s, 63°C for 30 s and 72°C for 1 min. These PCR reactions were run on a 1.5% agarose gel, with positive colonies running at 520 bp. Positive colonies were mini prepped (Qiagen mini prep kit, used according to manufacturers' directions) before Sanger sequencing through Source BioScience, using the protocol for strong hairpin structures. Miniprepped vectors that showed correct insertion of our shRNA sequence were selected for midiprep (Qiagen) and restriction digest with BamHI to check vector fragment size.

#### Vector digestion

Briefly, the psOPTIkd vector (kindly supplied by Ludovic Vallier laboratory, Wellcome Trust-Medical Research Council Cambridge Stem Cell Institute) was digested using restriction enzymes BglII and SalI (Thermo Fisher Scientific) in FastDigest buffer (Thermo Fisher Scientific) for 30 min at 37°C to allow insertion of different shRNA sequences against BNC1. The digested vector product was purified with the QIAquick PCR purification kit (Qiagen) and run on a 0.8% agarose gel before extraction using the QIAEX II Gel Extraction Kit (Qiagen).

#### Gene targeting by lipofection

For psOPTiKD of hPSC, AAVS1 targeting was performed by lipofection. hPSCs were transfected 24-48 h after cell passaging with 4 μg of DNA and 10 μl per well of Lipofectamine 2000 in Opti-MEM media (Gibco), according to the manufacturer's instructions. Briefly, cells were washed twice in PBS before incubation at RT for up to 45 min in 1 ml OptiMEM (Gibco). While cells were incubated in OptiMEM, DNA-OptiMEM mixtures were prepared. Mix 1 comprised 4 μg DNA (equally divided between the two AAVS1 ZFN plasmids, a kind gift from Ludovic Vallier laboratory, and our shRNA targeting vector) in 250 μl OptiMEM per well of a six-well plate. Mixture 2 comprised 10 μl Lipofectamine in 250 μl OptiMEM per well. Mixtures 1 and 2 were prepared and mixed gently before incubation at RT for 5 min. Then 250 μl of Mixture 2 was added to 250 μl Mixture 1 before incubation at RT for 20 min. Five hundred microliters of a 1:1 Mixture 1:Mixture 2 transfection mix was added in a drop-wise spiral manner around the well of hPSCs. Cells were incubated in transfection mix at 37°C overnight before washing in CDM-BSA II media the next day approximately 18 h post-transfection. After 2 days, 1 μg ml^−1^ of puromycin was added to the CDM-BSA II culture media. Individual hPSC clones were picked and expanded in culture in CDM-BSA II following 7-10 days of puromycin selection.

#### Genotyping siKD hPSC clones

Clones from gene targeting were screened by genomic PCR to verify site-specific targeting, determine whether allele targeting was heterozygous or homozygous, and check for off-target integrations of the targeting plasmid. (See Table S6 for PCR primers and thermocycling conditions and Fig. S6 for PCR results.) All PCRs were performed using 100 ng of genomic DNA as template in a 25 μl reaction volume using LongAmp Taq DNA Polymerase (NEB) according to the manufacturer’s instructions, including 2.5% volume dimethyl sulphoxide (DMSO). DNA was extracted using the genomic DNA extraction kit from Sigma-Aldrich according to the manufacturer’s instructions.

#### Inducible BNC1 knockdown

One homozygous-targeted clone for each vector transfection was selected for subsequent differentiation into hPSC-epi with or without the addition of 1 μg/ml tetracycline (Sigma-Aldrich) to culture media with the aim of mediating *BNC1* knockdown. hPSC-epi was successfully differentiated from each clone in the presence and absence of tetracycline. qPCR analysis indicated that clone 1Ei had a very pronounced reduction in *BNC1* (Fig. S7A). Another clone was generated with the vector BNC1-E (1E17) and this showed the same level of efficiency at downregulating BNC1 (Fig. S7B).

### Retroviral transduction

#### Production of the lentiviral particles

The lentiviral particle supernatant was obtained from transfection of 293T cells with the lentiviral vector of interest using Mirus *Trans*IT-LT1 transfection reagent and the HIV-1 helper plasmid psPAX2 (Addgene #12260) and HIV-1 envelope plasmid pMD2.G (Addgene #12259).

#### Production of fluorescent hPSC lines

While splitting, the H9 cells were transduced with a lentivirus expressing an EGFP reporter under the control of Ef-1α (*EEF1A1*) promoter. We used the lentiviral vector pLVTHM (Addgene #12247).

### Immunofluorescence

Primary antibodies were as follows: unconjugated or Alexa Fluor 488-conjugated rabbit anti-WT1 [CAN-R9(IHC)-56-2] (Abcam, ab89901 or ab202635; 1/100); rabbit anti-BNC1 (Atlas Antibodies, HPA063183; 1/200); rabbit anti-TCF21 (Atlas Antibodies, HPA013189; 1/100); mouse anti-THY1 clone 5E10 (Thermo Fisher Scientific, 14-0909-82; 1/100); mouse Anti-CNN1 (Sigma-Aldrich, C2687; 1/1000); rabbit anti-periostin (Abcam, ab14041; 1/500); mouse anti-synaptotagmin 4 (Abcam, ab57473; 1/100).

#### Cultured cells

Cells were fixed using 4% paraformaldehyde (PFA), permeabilised and blocked with 0.5% Triton X-100/3% BSA/PBS for 60 min at RT. Unless otherwise stated, primary antibody incubations were performed at 4°C overnight and Alexa Fluor-tagged secondary antibodies (A21200, A10037, A21238, A11034, A10042 and A21244, Invitrogen) were applied for 1 h at RT. Nuclei were counterstained with DAPI (10 μg/ml, Sigma-Aldrich).

For double staining of TCF21/WT1 and BNC1/WT1, TCF21 (or BNC1) and WT1 were detected sequentially. Anti-TCF21 or anti-BNC1 were first applied overnight and detected with a Rhodamin-FAB fragment goat anti-rabbit IgG (H+L) from Abcam for 1 h at RT. Then, the anti-WT1 conjugated to Alexa Fluor 488 was incubated for 2 h at RT. For double staining of THY1/WT1 or THY1/BNC1, the cells were first blocked without permeabilisation and THY1 was first detected with mouse anti-THY1 followed by incubation with anti-mouse conjugated antibody. The cells were briefly post-fixed in PFA 4% and then permeabilised with 0.5% Triton X-100/3% BSA/PBS before being incubated as for anti-WT1 or anti-BNC1.

Images were acquired on a Zeiss LSM 700 confocal microscope and analysed with ImageJ software. For quantification of the number of GFP^+^ cells also positive for CF or SMC markers, the number of GFP^+^ cells was first measured in the green channel. Then the double-positive or double-negative (depending on the size of the populations) cells were counted using the merge images. When few GFP^+^ cells were present in the image, the cells were manually counted using the Analyse ImageJ plug-in called ‘cell counter’ (https://imagej.nih.gov/ij/plugins/cell-counter.html). When too many GFP^+^ cells were present to count manually, we used the function ‘Analyse particles’ after adjustment of the threshold and binary transformation of the image. The double-positive or double-negative cells were counted using ‘cell counter’.

#### Cryostat sections

Foetal human hearts were harvested as described for primary human culture of epicardium. The whole heart was snap-frozen in liquid nitrogen and stored at −80C before sectioning in a cryostat after embedding in OCT; 10 µm-thick sections were collected onto SuperfrostPlus slides and stored at −80°C until staining. Staining was performed as described above.

### THY1 flow cytometry

Each sample of 10^6^ cells was divided into two tubes. One tube received a mouse isotype control antibody and the other tube was incubated with the mouse anti-THY1 antibody, clone 5E10 (both at 5 μg/ml final concentration) for 1 h at RT. After a rinse in 1× PBS, the cells were re-suspended in chicken anti-mouse Alexa 488 or donkey anti-mouse Alexa 647 antibody diluted 1/500.

### Quantitative real-time polymerase chain reaction

Total RNA was extracted using the RNeasy mini kit (Qiagen). cDNA was synthesised from 250 ng RNA using the Maxima First Strand cDNA Synthesis kit (Fermentas). Quantitative real-time polymerase chain reaction (qRT-PCR) reaction mixtures were prepared with SYBR green PCR master mix (Applied Biosystems) and run on the 7500 Fast Real-time PCR system using the quantitative relative standard curve protocol against standards prepared from pooled cDNA from each experiment. Melt curves were checked for each experimental run. CT values were normalised to housekeeper genes porphobilinogen deaminase (*PBGD*) or *GAPDH*. Primers were supplied by Sigma-Aldrich and sequences were as follows: *GAPDH* forward: AACAGCCTCAAGATCATCAGC; *GAPDH* reverse: GGATGATGTTCTGGAGAGCC; *WT1* forward: CACAGCACAGGGTACGAGAG; *WT1* reverse: CAAGAGTCGGGGCTACTCCA; *TCF21* forward: TCCTGGCTAACGACAAATACGA; *TCF21* reverse: TTTCCCGGCCACCATAAAGG; *BNC1* forward: GGCCGAGGCTATCAGCTGTACT; *BNC1* reverse: GCCTGGGTCCCATAGAGCAT.

### Western blotting

To assess BNC1 levels by immunoblotting, lysate from one confluent well of hPSC-epi cells in a six-well plate was separated by SDS-PAGE on an 8% acrylamide gel and transferred overnight onto a PVDF membrane. The protein was detected using a rabbit anti-BNC1 antibody (HPA063183, Atlas Antibodies; 1/100) followed by chemiluminescence detection using an HRP-conjugated secondary antibody, (7074S, NEB; 1/10,000). Mouse anti-β-tubulin antibody (86298, Cell Signaling Technology; 1/1000) was used as the housekeeping protein.

### BNC1 siRNA-mediated knockdown in human primary foetal epicardial cells

siRNA-mediated knockdown was performed by transfection via Optimem (Gibco) and Dharmafect (Dharmacon), and siRNA assays s2011 and s57438 against BNC1 (Thermo Fisher Scientific) as per the manufacturer’s directions, with a scrambled siRNA sequence (AllStar) and no siRNA as controls. Final siRNA concentration was 40 nm.

## Supplementary Material

Supplementary information
