# Supplementary material for: BNC1 regulates cell heterogeneity in human pluripotent stem cell-derived epicardium
Source: Development. 2019 Dec 13;146(24):dev174441. doi: 10.1242/dev.174441 (PMC6955213; doi:10.1242/dev.174441)
Supplement: Supplementary information [file develop-146-174441-s1.pdf]

SUPPLEMENTARY FIGURES

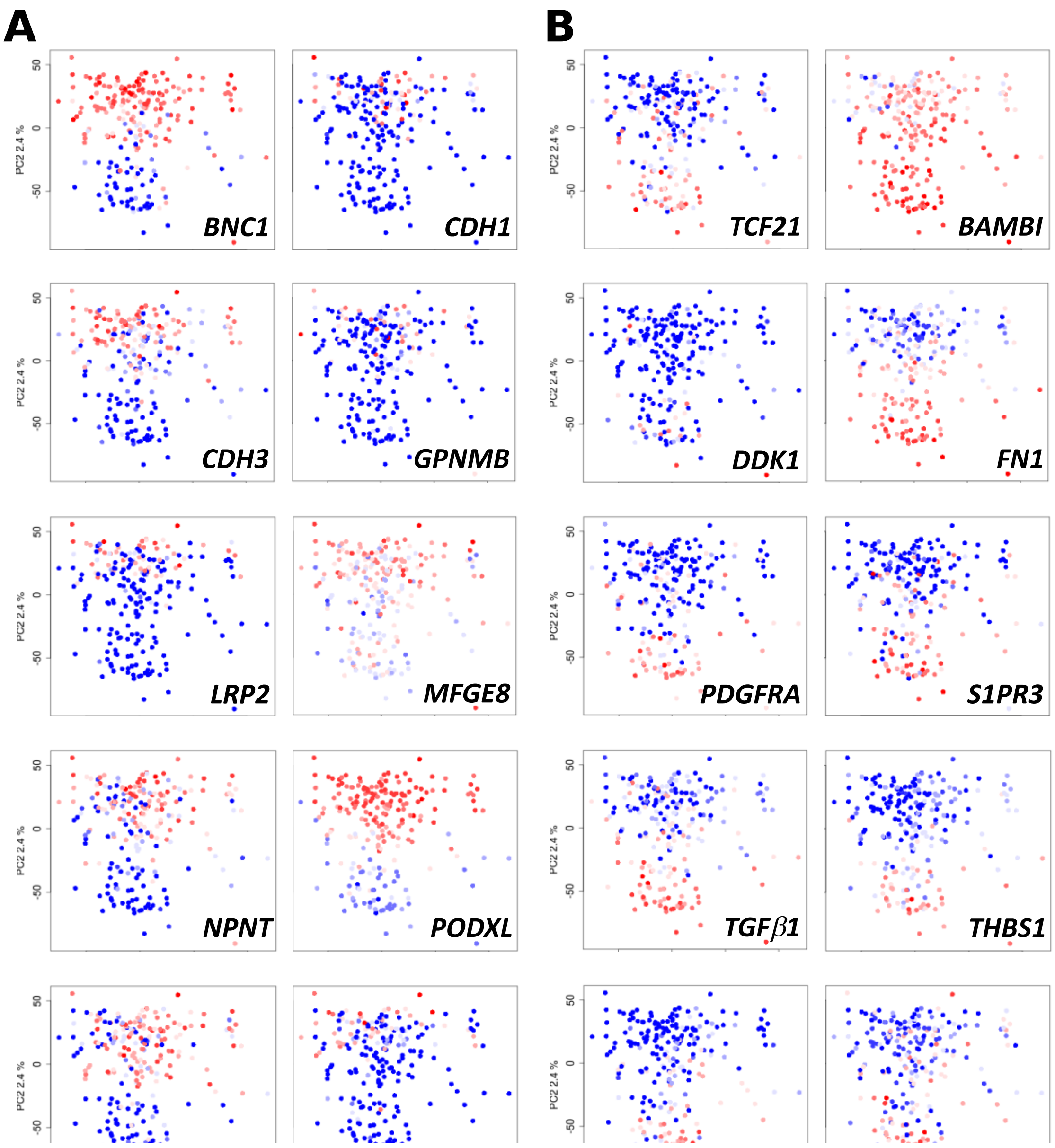

Figure S1: Expression of selected genes characteristic of the two subpopulations. Principal component analysis of the epicardial cells, coloured by the expression of selected genes. A) Genes expressed mainly in BNC1high cells. B) Genes expressed mainly in TCF21cells.

Development • Supplementary information

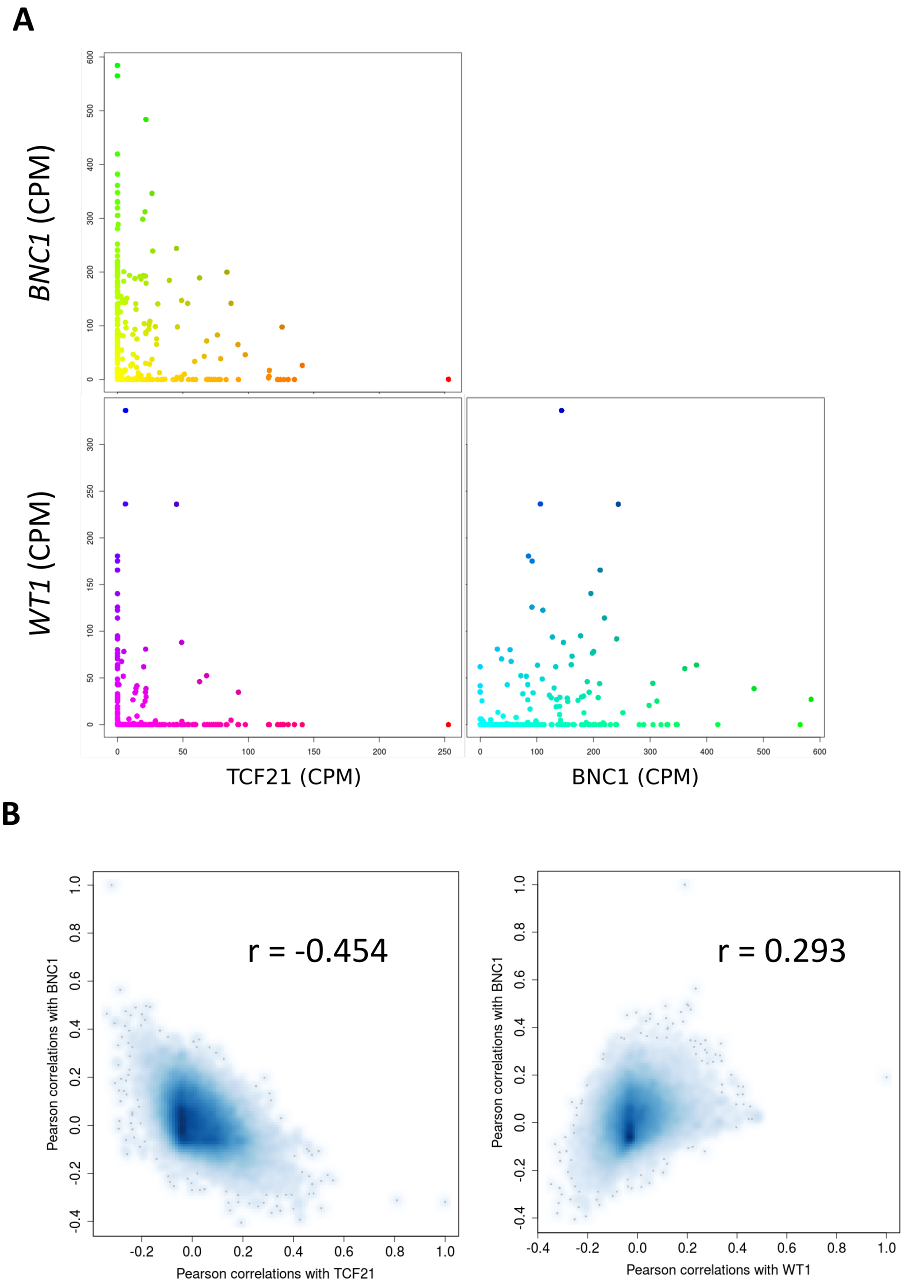

Figure S2: BNC1 expression correlates with WT1 and anti-correlate with TCF21.

A) Comparison of expression (in counts per millions reads) between BNC1, WT1 and TCF21 in all cells. Cells aligned to the axes have 0 counts for one of the genes, many of them probably dropouts. For non-0 expressions, cells expressing high levels of BNC1 tend to express high levels of WT1 and low levels of TCF21. B) Comparisons between Pearson correlations of BNC1, WT1 and TCF21 with all other genes. If a gene expression correlates with that of BNC1, it tends to anti-correlate with that of TCF21 but correlate with that of WT1.

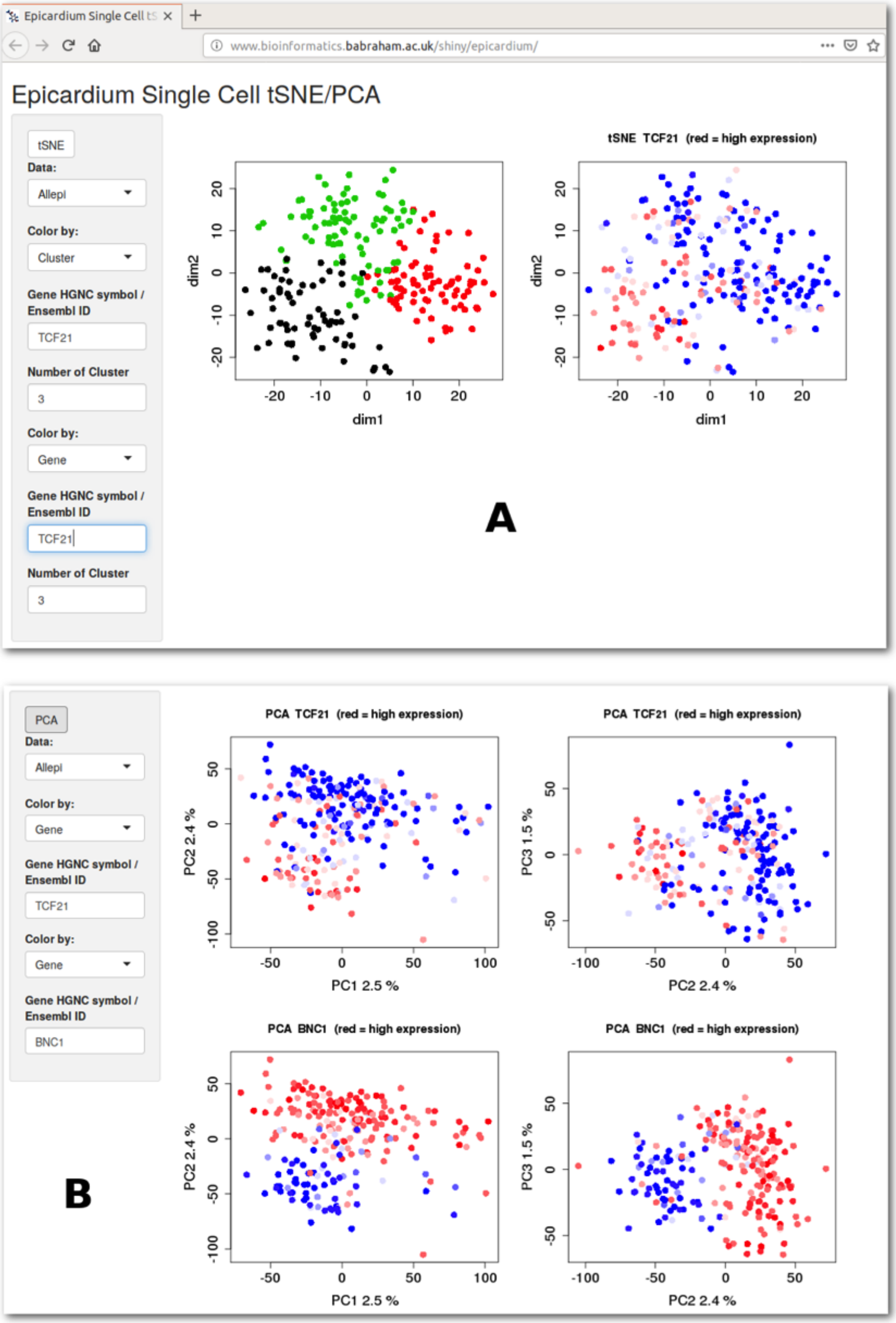

Development • Supplementary information

Figure S3: Web application to visualise our scRNAseq dataset

This web application developed with R's Shiny package can be found at https://www.bioinformatics.babraham.ac.uk. It is formed of two parts. A) Two tSNE plots, B) Two sets of PCA plots, comparing PC1 and PC2 (left), or PC2 and PC3 (right).

The procedure is the same for both parts. Firstly, the user must click the top button (re: "tSNE" or "PCA"). The cells are then plotted in black. One can change the dataset plotted between "Allepi", containing only epicardial cells and "All", containing epicardial and lateral mesoderm cells. Cells can be colored in black ("none"), according to the 96 plates they comes from, or with the expression of a particular gene. Genes have to be specified using HUGO gene symbols.

In addition, tSNE plots can be colored with clusters, the number of clusters being chosen by the user. The distribution of the expression for a gene of interest can thus be compared with the different clusters. In the example shown, we asked to distribute cells in three clusters in the tSNE and to colour the cells for TCF21 expression (in A) and colored the PCA with the expression of TCF21 and BNC1 (B).

Development • Supplementary information

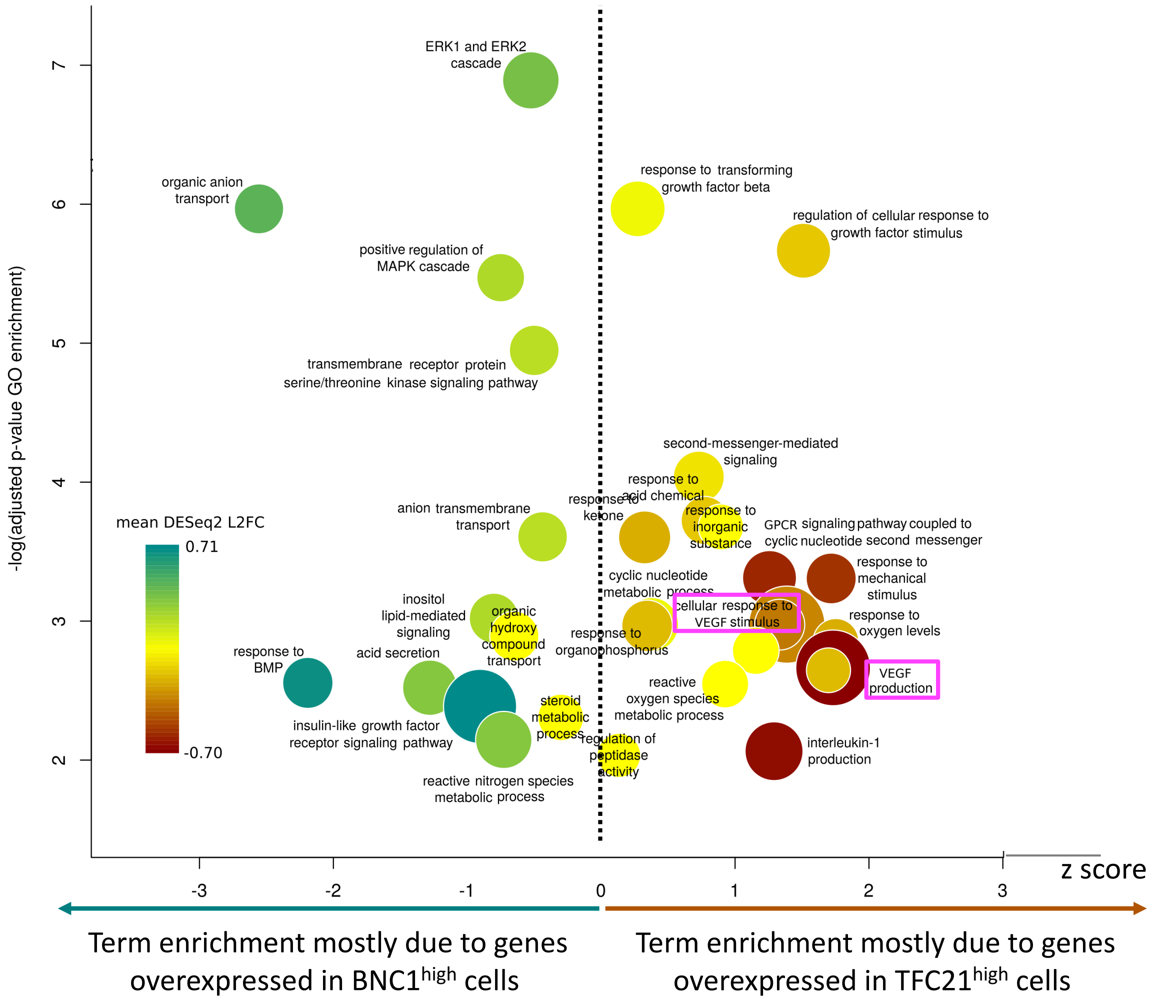

Figure S4: Predicted molecular process specificities of BNC1$_{high}$ and TCF21$_{high}$ cells

Results of Gene Ontology over-representation and gene expression differential analyses. Each bubble represents an over-represented GO term, the disk size being proportional to the enrichment. The vertical axis presents the significance of the enrichment while the horizontal axis indicates if the term enrichment is mostly due to genes over-expressed in BNC1$_{high}$ cells (negative z-scores) or in TCF21$_{high}$ cells (positive z-scores). Bubble colors show the mean difference of expression, for all the genes annotated by the GO term, between BNC1$_{high}$ cells (turquoise) and TCF21$_{high}$ cells(magenta) the two populations.

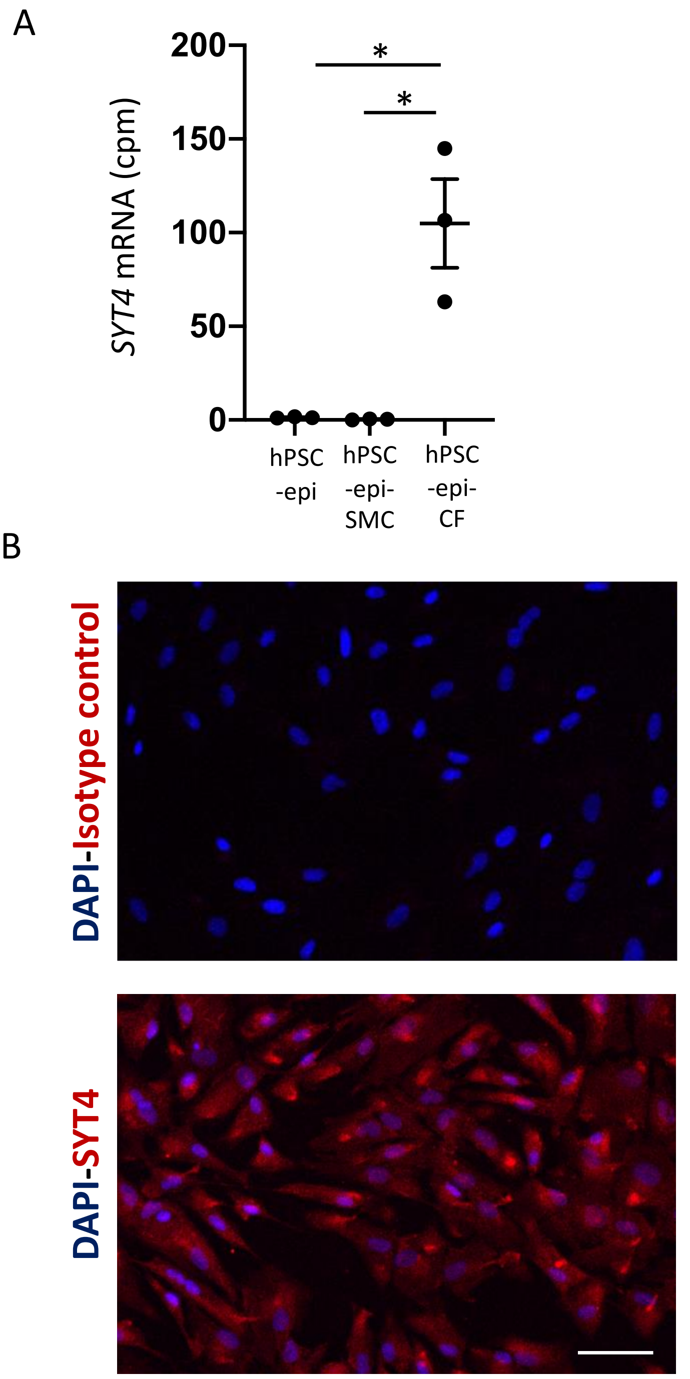

Figure S5 : SYT4 is a marker of hPSC-epi CF and is expressed in human foetal cardiac fibroblasts

(A) Expression of SYT4 mRNA in counts per million in hPSC-epi, hPSC-epi-SMC and hPSC-epi-CF (n=3 for each type). Error bars are s.e.m. Data were analysed with ratio paired t-test performed in Prism 7 from GraphPad. **(B)** Detection of SYT4 by immunocytochemistry in human foetal cardiac fibroblasts derived from 8 weeks pc human heart. Scale bar = 65µm

Development • Supplementary information

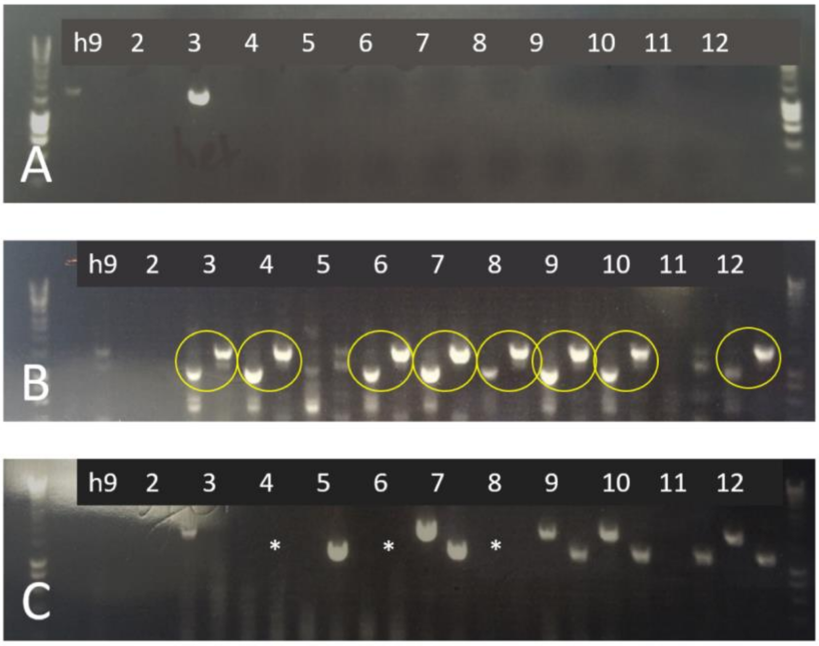

Figure S6: Representative gels for the genotyping of siKD clones

(A) Representative blot for homozygous targeted clones; WT H9 give a band at 1692 bp whereas no band indicates homozygous transgene integration. In this gel, all clones except ‘3’ appear to be homozygous-targeted. **(B)** 5’ and 3’ vector integration representative gel. 5’ integration is shown by a gel band at 1103bp whereas 3’ integration of insert is represented by a band at 1447bp. In this example, clones 3, 4, 6, 7, 8, 9, 10 and 12 appear to have 5’ and 3’ integration (circled). **(C)** Representative blot for off-target vector backbone integration into genomic DNA. In this gel, bands indicate off-target plasmid integration, whereas a lack of band indicates no off-target integration. Clones 4, 6 and 8 appear to be free from off-target integration (indicated by asterisks).

Development • Supplementary information

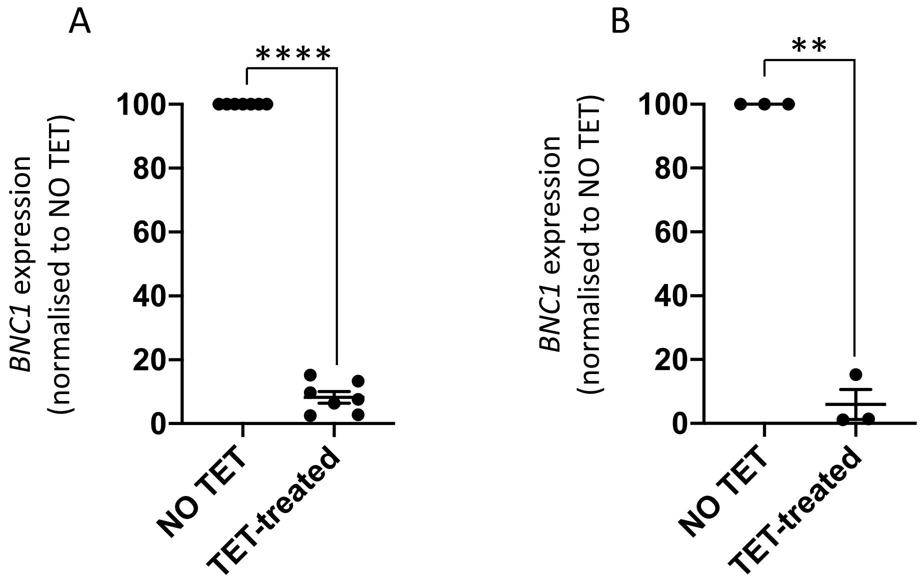

Figure S7: Hairpin 'E' mediate reduction in BNC1 mRNA after 9 days of tetracycline application. Clones '1Ei' (A, n=7 differentiation) and '1E17' (B, n=3 differentiation) have strong reduction of *BNC1* mRNA after application of tetracycline during lateral plate mesoderm and all along the epi differentiation. Statistics were performed with Prism 7 from GraphPad with a ratio paired t-test. Error bars = s.e.m

Development • Supplementary information

SUPPLEMENTARY TABLES

Table S1: Gene Ontology over-representation analysis of the genes differentially expressed between clusters TCF21$_{high}$, BNC1$_{high}$ and "mixed". Rows are enriched GO terms. Columns: "Geneset": GO term identifiers; "description": GO term description; "link": URL to and online version of the GO term; "C": total number of genes annotated by this GO term in the reference (background) list; "O": observed number of genes in the analysed gene list; "E": expected number of genes in the analyses gene list; "R": enrichment for the GO term (O/E); PValue: significance of the enrichment from an hypergeometric test; "FDR": False Discovery Rate (a.k.a adjusted p-value) after Benjamini-Hochberg correction; "overlapGene": index of the enriched genes in the submitted gene list; "OverlapGene_UserID": symbols of the enriched genes in the analysed gene list.

Click here to Download Table S1

Table S2: Differential Expression analysis between clusters BNC1$_{high}$ and TCF21$_{high}$. Rows are genes. Columns: "baseMean": normalised mean of gene raw counts between all samples; "log2FoldChange": extend of the difference of gene expression between the two clusters; "lfcSE": Standard Error of the log2FoldChange; "Stat": Wald statistics (L2FC/lfcSE); "pvalue": significance of the differential expression (Wald statistics compared to normal distribution); "padj": p-value after Benjamini-Hochberg correction for multiple testing.

Click here to Download Table S2

Table S3: Gene Ontology over-representation analysis of the genes differentially expressed between clusters TCF21$_{high}$ and "BNC1$_{high}$". See Supplementary Table 2 for an explanation of the columns. This is the full table, before removal of irrelevant terms, and without separation between terms pertaining to tissular and molecular processes.

Click here to Download Table S3

Development • Supplementary information

Table S4: List of the 100 top influences between one of {BNC1, TCF21, WT1} and a transcription factor. Only 297 influences are listed since 3 were present twice. Columns: “regulatory”: origin of the influence; “target”: gene which is influenced. “weight_CLR”: weight of the influence predicted by CLR; “rank_CLR”: rank of the influence in the list predicted by CLR (“1” would be the strongest predicted influence); “weight_GENIE3”: score of the influence predicted by GENIE3; “rank_GENIE3”: rank of the influence in the list predicted by GENIE3; “combined_weight”: product of “weight_CLR” and “weight_GENIE3”; “combined_rank”: rank of the predicted influence, as scored by the “combined_weight”.

Click here to Download Table S4

Table S5: shRNA sequences for psOPTIkd vector construction. BglII overhang is in red, terminator sequence/SalI overhang in blue. Hairpin loop sequence is in bold. Stem is underlined.

Gene/ shRNA ID	Top oligonucleotide (5' to 3')	Bottom oligonucleotide (5' to 3')
B2M – A	GATCCCGGACTGGTCTTTCTATCTC**TTCAAGAGA**GAGATAGAAAGACCAGTCCTTTTTTG	TCGACAAAAAAGGACTGGTCTTTCTATCTCT**CTCTTGAA**GAGATAGAAAGACCAGTCCGG
BNC1 - B	GATCCCGCCACACCATTTCAGGTTGAAA**CTCGAG**TTTCAACCTGAAATGGTGTGGTTTTTTG	TCGACAAAAAACCACACCATTTCAGGTTGAAA**CTCGAG**TTTCAACCTGAAATGGTGTGGCGG
BNC1 – C	GATCCCGCCTTGTCTTGAAGATTCTAAA**CTCGAG**TTTAGAATCTTCAAGACAAGGTTTTTTG	TCGACAAAAAACCTTGTCTTGAAGATTCTAAA**CTCGAG**TTTAGAATCTTCAAGACAAGGCGG
BNC1 – D	GATCCCGCCTGCACTGATAGGGTCATTG**CTCGAG**CAATGACCCTATCAGTGCAGGTTTTTTG	TCGACAAAAAACCTGCACTGATAGGGTCATTG**CTCGAG**CAATGACCCTATCAGTGCAGGCGG
BNC1 - E	GATCCCGGAGAGTAGTGAAGATCATTT**CTCGAG**AAATGATCTTCACTACTCTCCTTTTTTG	TCGACAAAAAAGGAGAGTAGTGAAGATCATTT**CTCGAG**AAATGATCTTCACTACTCTCCGG

Table S6: PCR primer sequences and cycling conditions for genotyping psOPTIkd clones

PCR type	Primer sequence	Primer name	Amplicon wild-type	Amplicon transgene integration	Amplicon plasmid integration	Annealing temperature degrees C	Extension time
Locus ('1')	CTGTTTCCCCTTCCCAGGCAGGTCC	AAVS1_Genomic_FOR	1692	Variable	No band	65	over 3 minutes
Locus ('1')	TGCAGGGGAACGGGGCTCAGTCTGA	AAVS1_Genomic_REV					
5' integration into AAVS1('2')	CTGTTTCCCCTTCCCAGGCAGGTCC	AAVS1_Genomic_FOR	No band	1103	No band	65	1'30
5' integration into AAVS1 ('2')	TCGTCGCGGGTGGCGAGGCGCACCG	AAV Pur_REV					
3' integration into AAVS1 ('3')	CCACCGAGAAGCAGTACGAG	OPT-tetR_For	No band	1447	No band	60	1'30
3' integration into AAVS1 ('3')	TGCAGGGGAACGGGGCTCAGTCTGA	AAVS1_Genomic_R					
5' off-targets ('4')	ATGCTTCCGGCTCGTATGTT	AAVS1_5'BB_For	No band	No band	1227	60	1'30
5' off-targets ('4')	TGAGGAAGAGTTCTTGCAGCTC	AAVS1_5'BB_Puro_Rev					
3' off-targets ('5')	CCACCGAGAAGCAGTACGAG	OPT-tetR_For	No band	No band	1802	60	2'
3' off-targets ('5')	ATGCACCACCGGGTAAAGTT	AAVS1_3'BB_Rev					
